# *STAT3* and *STAT5B* Mutations in T/NK-Cell Chronic Lymphoproliferative Disorders of Large Granular Lymphocytes (LGL): Association with Disease Features

**DOI:** 10.3390/cancers12123508

**Published:** 2020-11-25

**Authors:** Noemí Muñoz-García, María Jara-Acevedo, Carolina Caldas, Paloma Bárcena, Antonio López, Noemí Puig, Miguel Alcoceba, Paula Fernández, Neus Villamor, Juan A. Flores-Montero, Karoll Gómez, María Angelina Lemes, Jose Carlos Hernández, Iván Álvarez-Twose, Jose Luis Guerra, Marcos González, Alberto Orfao, Julia Almeida

**Affiliations:** 1 Translational and Clinical Research Program, Centro de Investigación del Cáncer and IBMCC (CSIC—University of Salamanca), Cytometry Service, NUCLEUS, Department of Medicine, University of Salamanca (USAL) and Institute of Biomedical Research of Salamanca (IBSAL), 37007 Salamanca, Spain; noemimg@usal.es (N.M.-G.); mariajara@usal.es (M.J.-A.); carolina.caldas@usal.es (C.C.); pabarcena@usal.es (P.B.); antuam@usal.es (A.L.); jflores@usal.es (J.A.F.-M.); orfao@usal.es (A.O.); 2 Biomedical Research Networking Centre Consortium of Oncology (CIBERONC), Instituto de Salud Carlos III, 28029 Madrid, Spain; noepuig@gmail.com (N.P.); alcocebasanchez@saludcastillayleon.es (M.A.); VILLAMOR@clinic.cat (N.V.); ivana@sescam.jccm.es (I.Á.-T.); margondi@usal.es (M.G.); 3 Hematology Service, University Hospital of Salamanca, Translational and Clinical Research Program, Centro de Investigación del Cáncer/IBMCC and IBSAL, 37007 Salamanca, Spain; 4 Institut für Labormedizin, Kantonsspital, 5001 Aarau, Switzerland; paula.fernandez@ksa.ch; 5 Department of Pathology, Hematopathology Unit, Hospital Clínic, IDIBAPS, 08036 Barcelona, Spain; 6 Hematology Service, Juan Ramón Jiménez Hospital, 21005 Huelva, Spain; karollgomezcorrecha@gmail.com; 7 Hematology Service, Dr. Negrín Hospital, 35010 Las Palmas de Gran Canaria, Spain; alemcas@gobiernodecanarias.org; 8 Hematology Service, Punta de Europa Hospital, Algeciras, 11207 Cadiz, Spain; hematologiahpe@gmail.com; 9 Instituto de Estudios de Mastocitosis de Castilla La Mancha (CLMast), Virgen del Valle Hospital, 45071 Toledo, Spain; 10 Hematology Service, Virgen de la Luz Hospital, 16002 Cuenca, Spain; jlguerra@sescam.jccm.es; 11 Department of Nursery and Physiotherapy, University of Salamanca, 37007 Salamanca, Spain

**Keywords:** large granular lymphocytic leukemia, *STAT3* and *STAT5B* mutations, T and NK cells, neutropenia, normal leukocyte subsets

## Abstract

**Simple Summary:**

*STAT3* and *STAT5B* mutations have been identified in a subset of T and NK large granular lymphocytic leukemia (T/NK-LGLL). The aim of our study was to evaluate the frequency and type of these mutations in all different subtypes of T/NK-LGL expansions (*n* = 100 patients), as well as to analyze its association with biological and clinical features of the disease. We show for the first time that *STAT3/5B* mutations were present in all different T/NK-cell LGLL categories here studied; further, *STAT3* mutations were associated with overall reduced counts of almost all normal residual populations of immune cells in blood, together with a shorter time-to-therapy vs. wild type T/NK-LGLL. These findings contribute to support the utility of the *STAT3* mutation analysis for diagnostic and prognostic purposes in LGLL.

**Abstract:**

*STAT3* and *STAT5B* (*STAT3/STAT5B*) mutations are the most common mutations in T-cell large granular lymphocytic leukemia (T-LGLL) and chronic lymphoproliferative disorders of NK cells (CLPD-NK), but their clinical impact remains unknown. We investigated the frequency and type of *STAT3/STAT5B* mutations in FACS-sorted populations of expanded T/NK-LGL from 100 (82 clonal; 6 oligoclonal; 12 polyclonal) patients, and its relationship with disease features. Seventeen non-LGL T-CLPD patients and 628 age-matched healthy donors were analyzed as controls. *STAT3* (*n* = 30) and *STAT5B* (*n* = 1) mutations were detected in 28/82 clonal T/NK-LGLL patients (34%), while absent (0/18, 0%) among oligoclonal/polyclonal LGL-lymphocytosis. Mutations were found across all diagnostic subgroups: TCD8^+^-LGLL, 36%; CLPD-NK, 38%; TCD4^+^-LGLL, 7%; Tαβ^+^DP-LGLL, 100%; Tαβ^+^DN-LGLL, 50%; Tγδ^+^-LGLL, 44%. *STAT3*-mutated T-LGLL/CLPD-NK showed overall reduced (*p* < 0.05) blood counts of most normal leukocyte subsets, with a higher rate (vs. nonmutated LGLL) of neutropenia (*p* = 0.04), severe neutropenia (*p* = 0.02), and cases requiring treatment (*p* = 0.0001), together with a shorter time-to-therapy (*p* = 0.0001), particularly in non-Y640F *STAT3-*mutated patients. These findings confirm and extend on previous observations about the high prevalence of *STAT3* mutations across different subtypes of LGLL, and its association with a more marked decrease of all major blood-cell subsets and a shortened time-to-therapy.

## 1. Introduction

Leukemia of large granular lymphocytes (LGLs) is a rare chronic lymphoproliferative disorder (CLPD) defined by an unexplained and persistent (>6 months) expansion (usually >2 × 10^9^/L) of clonal LGL cells in blood [1,2,3,4]. According to their normal counterpart, LGL leukemia (LGLL) is classified as T-cell LGLL (T-LGLL) or CLPD of natural killer (NK) cells (CLPD-NK) [3,4]. Most LGLL cases derive from TCRαβ^+^CD4^−^CD8^+^ T lymphocytes (TCD8^+^), less frequently from TCRαβ^+^CD4^+^CD8^−/+lo^ T cells (TCD4^+^), TCRγδ^+^ T cells (Tγδ^+^) or NK cells, and rarely from other cytotoxic cells (i.e., TCRαβ^+^CD4^-^CD8^−/+lo^ double-negative T cells -Tαβ^+^DN-) [4]. T-LGLL and CLPD-NK usually show an indolent clinical course [4,5], which is often associated with autoimmune conditions—e.g., cytopenias—and recurrent infections, that require therapeutic interventions [5,6,7,8,9,10]. In a few cases (<5%) transformation to aggressive leukemia has been reported [5,7,8,9,10,11].

At present, diagnosis of LGLL remains a challenge. This is mostly due to the absence in a significant fraction of patients of tumor-associated phenotypic and/or molecular markers of clonality that would allow clear cut distinction among expansions of clonal vs. reactive/oligoclonal T cells [6,12,13], together with the lack of an universal marker of clonality for NK cells [14]. In addition, clonal/oligoclonal LGLs are also frequently detected in blood of asymptomatic subjects, either transiently (i.e., after allogeneic hematopoietic stem cell transplantation or solid organ transplantation) or persistently (i.e., in the elderly) [9,10,15]. So far, no reliable predictors of (indolent vs. aggressive) disease behavior have been established for T-LGLL and CLPD-NK.

*STAT3* and *STAT5B* (signal transducer and activator of transcription 3 and 5B genes) activating somatic mutations have been identified in a subset of LGLL [16,17,18]. Thus, *STAT3* mutations have been reported in between 21% and 73% of TCD8^+^-LGLL [16,17,19,20,21,22,23,24,25,26] and in 13–70% of CLPD-NK [7,18,21,22,23,24,27], depending on the sensitivity of the approach used. In contrast, *STAT5B* mutations have been reported to be associated with TCD4^+^-LGLL (6/11 cases) [28], while rarely (<2%) detected in both TCD8^+^-LGLL and CLPD-NK [11,17,20,25]. Although *STAT3/5B* mutations present in LGLL are discussed in the WHO 2016 classification of hematological malignancies [4], its routine (clinical) utility as “clonal” markers of disease—particularly in cases showing expansion of cytotoxic T cells other than TCD8^+^-LGLL (i.e., Tγδ^+^-LGLL)—still remains to be established [18,19,20,21,24,26,29]. Similarly, the potential impact of *STAT3* mutations on disease behavior and patient outcome remains to be confirmed [18,26,30,31,32].

Here we investigated the frequency and type of somatic mutations of the *STAT3* and *STAT5B* genes in (purified) T- and NK-LGLs from a large series of T-LGLL and CLPD-NK patients. In addition, we analyzed the potential association between these gene mutations and both the lineage and biological features of clonal cells, the distribution of normal residual immune cells in blood, and other clinical features of the disease, including patient outcome.

## 2. Results

### 2.1. Frequency and Type of STAT3 and STAT5B SH2 Somatic Mutations in T/NK-LGLs

*STAT3* (*n* = 27) and *STAT5B* (*n* = 1) somatic mutations (located at the Src homology 2—SH2—domain at exons 20 and 21 and exon 16, respectively) were detected in 28/82 (34%) T/NK-LGL clonal cases and in 0/18 oligoclonal/polyclonal LGL lymphocytosis, for a total of 31/94 (33%) clonal LGL expansions investigated on FACS-purified suspicious T- and NK-cell populations (Table 1A); two distinct clonal LGL populations with a different *STAT3* mutation each, coexisted in three purified populations from patients (Table 1B). All mutations except one corresponded to heterozygous mutations (30/31, 97%), as they coexisted with wild-type gene sequences.

*STAT3* mutations were present at variable frequencies in all LGLL subgroups except TCD4^+^-LGLL. These included 4/7 (57%) CD56^−/lo^ CLPD-NK patients, 7/16 (44%) Tγδ^+^-LGLL, 12/33 (36%) TCD8^+^-LGLL, and 2/7 (29%) CD56^+^ CLPD-NK, in addition to the single TCRαβ^+^CD4^+^CD8^+^ (Tαβ^+^DP—double-positive) case analyzed, and 1/2 Tαβ^+^DN-LGLL patients studied (Table 1A). Among other previously described mutations, the *STAT3*-Y640F mutation was the most frequent (15/31 mutated-cell populations; 48%). Interestingly, it was present in all patient groups, except TCD4^+^-LGLL and CD56^+^ CLPD-NK, with variable frequencies (e.g., 60% of *STAT3*-mutated TCD8^+^-LGLL, 43% of *STAT3*-mutated Tγδ^+^-LGLL and 25% of *STAT3*-mutated CD56^−/lo^ CLPD-NK populations; Table 1B and Figure S1). In turn, the *STAT3* S614R mutation was detected in 2/7 purified Tγδ^+^-cell populations (29%), and the *STAT3*-D661V mutation was restricted to half (2/4) of the purified CD56^−/+lo^ CLPD-NK cell populations tested. Finally, a new *STAT3*-K658F mutation was identified in one FACS-sorted Tγδ^+^-cell population. The remaining *STAT3* mutations identified (G618R, N647I, G656ins, Y657dup, and D661Y) were distributed in the most frequent LGLL subtypes at variable frequencies (40% of TCD8^+^-LGLL, 14% of Tγδ^+^-LGLL, 25% of CD56^−/lo^, and 100% of CD56^+^ CLPD-NK populations). The only *STAT5B* mutation (Y665F) detected in our cohort corresponded to a TCD4^+^-LGLL patient (1/15; 7%) (Table 1B and Figure S1).

Of note, *STAT3*/*STAT5B* mutations were absent in all non-LGL T-CLPD patients, except for an adult T-cell leukemia/lymphoma case that showed the *STAT3*-S614R mutation. Similarly, none of the 26 purified polyclonal non-LGL lymphoid populations and the seven (FACS-sorted) myeloid-cell populations analyzed (collected from seven different patients with *STAT3*-mutated LGLs), tested positive for *STAT3*/*STAT5B* mutations.

### 2.2. Immunophenotype, Clonal Profile, and TCR-Vβ and TCR-VγVδ Repertoire of STAT3-Mutated vs. -Unmutated T/NK-LGLL Patients

**Figure 1:**
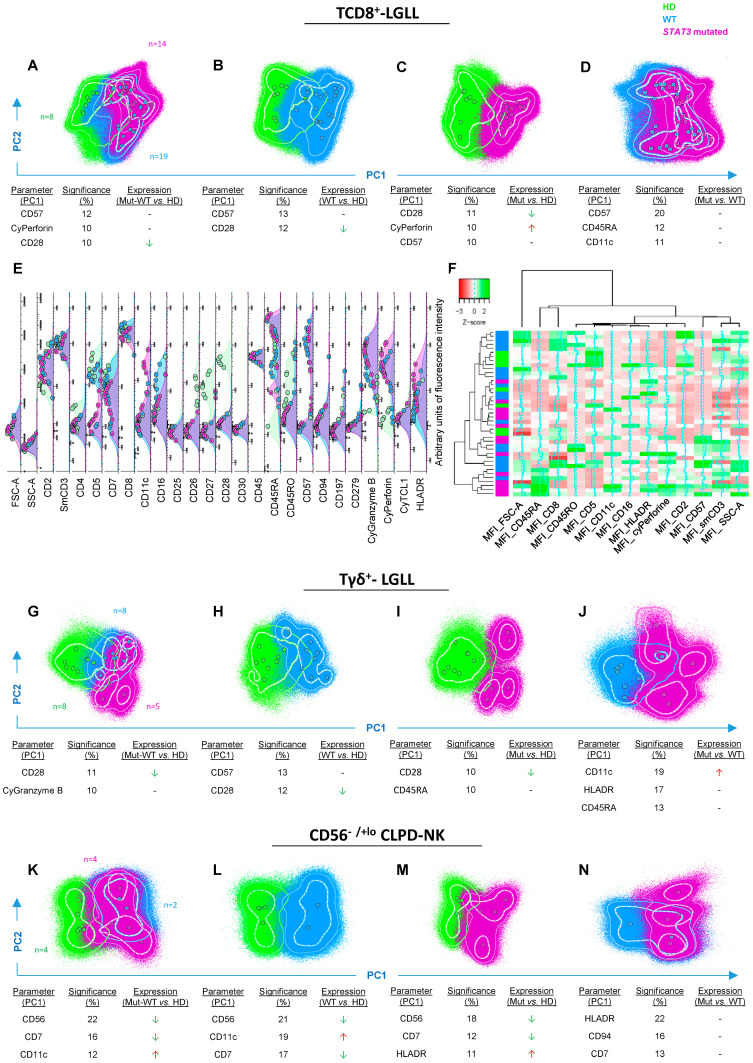
Phenotypic profiles of *STAT3*-mutated vs. WT clonal TCD8^+^-LGLL, Tγδ^+^-LGLL, and CD56^−/+lo^ CLPD-NK cell populations compared to their normal cell counterpart. Multidimensional phenotypic comparisons—principal component (PC) 1 vs. PC2, automatic population separator 1 (APS1) representations—showing those markers providing the best resolution (i.e., separation) between *STAT3*-mutated (depicted in purple) and WT (highlighted in blue) clonal LGL populations vs. normal cell populations (depicted in green). Overexpression (red arrows), underexpression (green arrows), or heterogeneous trend of expression (hyphen) of markers between the different set of cell populations. In all PC1 vs. PC2 (APS1) plots, solid circles represent median values for all phenotypic parameters evaluated, inner (dotted) and outer (solid) lines represent the first and the second SD for each population identified. (**A**): comparison between normal TCD8^+^-cell populations (*n* = 8) and both *STAT3*-mutated (*n* = 14) and WT (*n* = 19) clonal TCD8^+^-LGLL populations. (**B**): comparison between normal TCD8^+^-cell populations and WT clonal TCD8^+^-LGLL populations. (**C**): comparison between normal TCD8^+^-cell populations and *STAT3*-mutated clonal TCD8^+^-LGLL populations. (**D**): comparison between *STAT3*-mutated and WT clonal TCD8^+^-LGLL populations. (**E**): parameter band histogram of clonal *STAT3*-mutated (*n* = 14) and nonmutated (*n* = 19) clonal TCD8^+^-LGLL populations, as well as normal TCD8^+^ populations from adult healthy donors (*n* = 8) in which each solid circle represents median fluorescence intensity values per cell population. (**F**): heatmap representing hierarchical clustering analysis of the overall immunophenotype of clonal *STAT3*-mutated (*n* = 14) and nonmutated (*n* = 19) TCD8^+^-LGLL populations, compared to normal blood TCD8^+^ cells from adult healthy donors (*n* = 8). (**G**): comparison between normal Tγδ^+^ cells (*n* = 8) and both *STAT3*-mutated (*n* = 5) and WT (*n* = 8) clonal Tγδ^+^-LGLL populations. (**H**): comparison between normal Tγδ^+^ populations and WT clonal Tγδ^+^-LGLL populations. (**I**): comparison between normal Tγδ^+^ populations and *STAT3*-mutated clonal Tγδ^+^-LGLL populations. (**J**): comparison between *STAT3*-mutated and all WT clonal Tγδ^+^-LGLL populations. (**K**): comparison between normal blood CD56^+lo^ NK cells (*n* = 4) and both *STAT3*-mutated (*n* = 4) and WT (*n* = 2) clonal CD56^−/+lo^ CLPD-NK cell populations. (**L**): comparison between normal CD56^+lo^ NK-cell populations and WT clonal CD56^−/+lo^ CLPD-NK cell populations. (**M**): comparison of normal CD56^+lo^ NK cells vs. *STAT3*-mutated clonal CD56^−/+lo^ CLPD-NK cell populations. (**N**): comparison between *STAT3*-mutated and WT clonal CD56^−/+lo^ CLPD-NK cell populations. Abbreviation: Cy, cytoplasmic; HD, healthy donors; MFI, median fluorescence intensity; Mut, *STAT3*-mutated; WT, wild-type.

A high degree of overlap was observed in the global phenotype of *STAT3-*mutated vs. *STAT3* wild-type (WT) clonal TCD8^+^-cell populations, and their normal counterpart (Figure 1A–F). Despite this, differences between clonal and normal (total) TCD8^+^ cells were still evidenced, consisting of a more pronounced effector-cytotoxic phenotype for clonal vs. normal residual TCD8^+^ cells—i.e., higher levels of expression of CD57 (*p* = 0.003), cyGranzyme B (*p* = 0.00006), cyPerforin (*p* = 0.0001) and HLADR (*p* = 0.03) and lower amounts/cell of CD5 (*p* = 0.0002), CD7 (*p* = 0.0001), CD26 (*p* = 0.00007), CD27 (*p* = 0.00005), CD28 (*p* = 0.00002), CD45RO (*p* = 0.006), and CD197 (*p* = 0.003) in the former cells (Figure 1A–E). Similarly, the overall immunophenotype of *STAT3*-mutated TCD8^+^-LGL populations also overlapped partially with that of WT TCD8^+^ clonal LGL (Figure 1D,F), although *STAT3-*mutated cells tended to show higher percentages of effector-cytotoxic and activated cells, as reflected by higher expression levels of CD11c (*p* = 0.001), CD45RA (*p* = 0.03), HLADR (*p* = 0.009), and cyPerforin (*p* = 0.09), together with lower amounts of CD8 (*p* = 0.01) (Figure 1E,F).

Among Tγδ^+^-LGL, *STAT3-*mutated and -WT clonal Tγδ^+^ cells showed a more effector-cytotoxic and activated phenotype, associated with higher expression of CD57 (*p* = 0.009), cyGranzyme B (*p* = 0.02) and cyPerforin (*p* = 0.005), and lower amounts of CD5 (*p* = 0.0005), CD26 (*p* = 0.007), CD27 (*p* = 0.0002), CD28 (*p* = 0.0002), and CD45RO (*p* = 0.0003), vs. normal Tγδ^+^ cells (Figure 1G–I). Compared to WT (clonal) Tγδ^+^ cells, all five *STAT3*-mutated Tγδ^+^ clonal cell populations showed significantly higher expression of CD11c (*p* = 0.03; Figure 1J and Figure S2).

In contrast with TCD8^+^-LGL and Tγδ^+^-LGL, WT and *STAT3*-mutated CD56^−/+lo^ clonal NK cells showed a clearly distinct phenotype from normal CD56^+lo^ NK cells, due to lower expression of CD7 (*p* = 0.01) and CD56 (*p* = 0.01), and overexpression of CD11c (*p* = 0.01) (Figure 1K–M), particularly among WT CD56^−/+lo^ NK cells (*p* = 0.06 vs. *STAT3*-mutated cells; Figure 1N). In addition, *STAT3*-mutated CD56^−/+lo^ NK cells showed higher amounts/cell of HLADR vs. normal CD56^+lo^ NK cells (*p* = 0.02; Figure 1M). In turn, a high degree of overlap was observed between *STAT3*-mutated and nonmutated clonal CD56^−/+lo^ NK cells, except for 1/4 *STAT3*-mutated NK-cell populations, that showed a clearly different and unique phenotype vs. WT clonal NK cells (Figure 1N and Figure S2).

Preliminary results, for the remaining—very rare—LGLL groups (TCD4^+^-LGLL, Tαβ^+^DN-LGLL and CD56^+^ CLPD-NK), showed that the immunophenotype of the single *STAT3/STAT5B*-mutated population per group did not overlap with their WT counterpart (Figure S2).

Around one quarter (24%) of clonal LGL patients showed ≥2 coexisting clonal LGL populations, one of which systematically corresponded to TCD8^+^-LGLs. The overall frequency of *STAT3*-mutated monoclonal—21/30 cases (70%)—vs. biclonal—9/30; (30%)—cases investigated was similar (43% vs. 56% mutated cases, respectively; *p* > 0.05). Likewise, no clear association was found between the presence vs. absence of *STAT3* mutations and the TCR-Vβ or TCR-Vγ9Vδ2 repertoire of Tαβ-LGLL and Tγδ-LGLL cases, respectively (Table S1).

### 2.3. Distribution of Normal Residual PB Leukocyte Subsets and STAT3-Mutational Status in T/NK-LGLL

**Figure 2:**
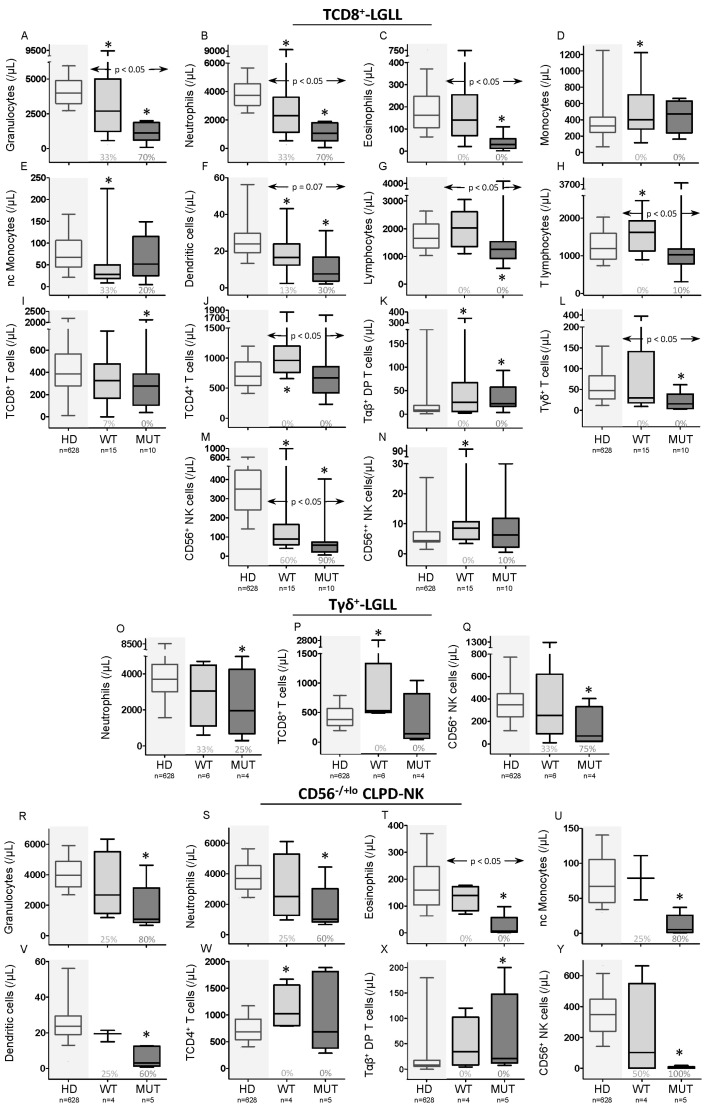
Distribution of normal peripheral blood (PB) leukocyte subsets in TCD8^+^-LGLL, Tγδ^+^-LGLL, and CLPD-NK patients classified according to their *STAT3* mutational status. (**A**–**N**): distribution of normal residual leukocyte subpopulations found to be significantly (*p*-value ≤ 0.05) different in PB in either WT (*n* = 15) and/or *STAT3*-mutated (*n* = 10) TCD8^+^-LGLL cases vs. age-matched HD (*n* = 628), and between *STAT3* WT and mutated TCD8^+^-LGLL cases. (**O**–**Q**): distribution of normal residual leukocyte subpopulations found to be significantly (*p*-value ≤ 0.05) different in PB of either WT (*n* = 6) and/or *STAT3*-mutated (*n* = 4) Tγδ^+^-LGLL cases vs. age-matched HD (*n* = 628), or between *STAT3*-WT and -mutated Tγδ^+^-LGLL cases. (**R**–**Y**): distribution of normal residual leukocyte subpopulations found to be significantly (*p*-value ≤ 0.05) different in PB of either WT (*n* = 4) and/or *STAT3*-mutated (*n* = 5) CLPD-NK cases vs. age-matched HD (*n* = 628), or between *STAT3*-WT and -mutated CLPD-NK cases. In the above panels, notched boxes represent 25th and 75th percentile values; the lines in the middle correspond to median values (50th percentile) and vertical lines represent 10th and 90th percentile. The numbers under the boxes indicate the percentage of cases that showed cell counts below the minimum value found among HD. The horizontal arrows mean the *p*-value between WT and *STAT3*-mutated cases. * *p*-value ≤ 0.05 vs. HD. Abbreviations (alphabetical order): HD, healthy donors; MUT, *STAT3*-mutated; nc, nonclassical; *p*, *p*-value; WT, wild-type.

Blood counts for most normal leukocyte subsets (i.e., neutrophils, eosinophils, dendritic cells, Tγδ^+^, and CD56^+^ NK cells) were significantly reduced (vs. age-matched HD) among T/NK-LGLL, particularly among *STAT3*-mutated T/NK-LGLL patients (Table 2). Thus, *STAT3-*mutated T/NK-LGLL patients showed significantly decreased (*p* ≤ 0.05 vs. age-matched HD) normal residual neutrophil, eosinophil, nonclassical monocyte, dendritic-cell, Tγδ^+^-cell, and CD56^+^ NK-cell counts (Table 2) in blood, independently of the type of *STAT3* mutation (i.e., both in Y640-mutated and non-Y640F-mutated cases), while a statistically significant decrease of B-cell counts was observed only among non-Y640F *STAT3*-mutated cases vs. age-matched HD: median (range) of 86 (15–251)/μL vs. 141 (8.1–867)/μL, respectively (*p* = 0.05); further, B-cell counts was also statistically significantly reduced in non-Y640F *STAT3*-mutated cases (*n* = 12) vs. Y640F-mutated cases (*n* = 13)—86 (15–251)/μL vs. 198 (105–342)/μL respectively (*p* = 0.02). In turn, WT T/NK-LGLL cases showed significantly reduced (vs. age-matched HD) neutrophil, eosinophil, nonclassical monocyte, dendritic cell, and CD56^+^ NK-cell counts in blood, in association with significantly higher basophil, total monocyte, and total lymphocyte counts (at the expense of TCD4^+^ and Tαβ^+^DP cells; Table 2). These differences in the distribution of normal blood-cell subsets among *STAT3* mutated (and to a lesser extent also WT T/NK-LGLL cases), were confirmed when T-LGLL and TCD8^+^-LGLL cases were separately analyzed (including the reduction of B cells only in non-Y640F *STAT3*-mutated cases), with additional decreased TCD8^+^-cell and Tγδ^+^-cell counts among *STAT3*-mutated total T-LGLL and TCD8^+^-LGLL cases, and increased CD56^++^ NK-cell numbers among WT TCD8^+^ cases (Figure 2A–N and Tables S2 and S3). Interestingly, all above referred normal T/NK-LGL subsets were similarly reduced in monoclonal vs. biclonal *STAT3*-mutated TCD8^+^-LGLL patients, except for Tγδ^+^ cells, which were decreased (*p* = 0.006) only among *STAT3*-mutated patients (Figure S3). In turn, among WT cases, reduced (*p* ≤ 0.05 vs. controls) counts of neutrophils, monocytes, dendritic cells, and NK cells were restricted to monoclonal cases, while biclonal patients showed normal or even increased—e.g., basophil (*p* = 0.02) and classical monocyte (*p* = 0.003)—counts in blood of these cell populations, compared to HD (Figure S3).

Upon comparing the distribution of normal residual T cells of TCD8^+^-LGLL cases into maturation stages, a similar distribution was observed in blood (Figure S4), except for naïve TCD4^+^-cell counts—median (range)—that were significantly reduced in both *STAT3*-mutated—260 (31–1013), *p* = 0.02—and unmutated—231 (45–755), *p* = 0.01—patients vs. HD—522 (160–1018) cells/µL.

Among Tγδ^+^-LGLL and CLPD-NK cases, a similar profile was observed (Tables S4 and S5) which consisted of decreased blood levels (vs. HD) of normal CD56^+^ NK cells (*p* = 0.04) of *STAT3*-mutated patients and increased normal TCD8^+^-cell counts (*p* ≤ 0.05) in WT Tγδ^+^-LGLL cases (Figure 2O–Q,Y). In turn, *STAT3*-mutated CLPD-NK had significantly reduced counts (vs. HD) of neutrophils, eosinophils, nonclassical monocytes, dendritic cells, and NK cells (at the expense of CD56^+^ NK cells), with values below the normal range in 60%–100% of patients. In contrast, WT CLPD-NK cases only showed a significant reduction of eosinophils in association with higher TCD4^+^-cell numbers in blood (Figure 2R–Y and Table S5).

### 2.4. Clinical Features of T/NK-cell LGLL with STAT3 and STAT5B Mutations vs. WT Cases

No statistically significant differences were detected between *STAT3*/*STAT5B*-mutated vs. WT LGLL patients as regards sex, age, presence of organomegalies or associated neoplasias other than the clonal T/NK-cell expansion(s). Likewise, a similar outcome (in terms of rate of progression or overall survival) was also observed between the two groups (Table 3). Despite this, statistically significant differences were observed between both groups of T/NK-LGLL patients as regards the frequency of neutropenia (32% vs. 13%, *p* = 0.04)—particularly of severe (<500 neutrophils/µL) neutropenia (14% vs. 0%, *p* = 0.02)—and the platelet count in blood (mean ± SD: 203 ± 88 vs. 243 ± 72 platelets × 10^9^/L respectively, *p* = 0.05), which were more frequently altered in *STAT3*-mutated vs. unmutated cases (Table 3), while hemoglobin levels were significantly reduced only in non-Y640F *STAT3*-mutated cases vs. WT cases (11 ± 2.2 vs. 13 ± 2.1 g/dL respectively, *p* = 0.001). In addition, *STAT3*-mutated T/NK-LGLL cases also required treatment more frequently (12/24, 50% vs. 4/44, 9%; *p* = 0.0001), due to the associated-autoimmune diseases (e.g., severe neutropenia); consequently, they also showed a significantly shorter time-to-(first) therapy after a median follow-up of 183 months: median (95% confidence interval) of 72 months (1–180) vs. not reached, respectively (*p* = 0.0001; Table 3), which was more evident in non-Y640F *STAT3*-mutated cases—2 months (1–6) vs. 93 months (59–127) in Y640F-mutated cases; *p* = 0.009. Of note, in one WT and one *STAT3*-Y640F mutated patient, the status of the mutation was investigated before and subsequently after therapy with very similar results. Similar clinical and laboratory findings were observed at presentation among T-LGLL cases (Table S6), but not when monoclonal vs. biclonal T-LGLL patients were compared (Table S7). Among CLPD-NK cases, *STAT3*-mutated patients also showed a significantly shorter time-to-therapy—6 months (1–15) vs. 88 months (56–119), *p* = 0.05, as well as lower levels of hemoglobin (*p* = 0.04) vs. WT cases, while the frequency of lymphocytosis in the latter patients was significantly higher than among *STAT3*-mutated CLPD-NK patients (100% vs. 50%, *p* = 0.05) (Table S8).

## 3. Discussion

Although *STAT3* and *STAT5B* mutations are present in a significant percentage of all LGLL patients, their clinical and biological significance remain to be (fully) established [18,19,20,21,24,26,29,31,33]. Here we confirm and extend on previous observations by showing a high frequency of *STAT3* mutations in both TCD8^+^-LGLL and CLPD-NK [16,17,18], but also among Tγδ^+^-LGLL and all other T/NK-lineage LGLL here studied. In addition, we also demonstrate for the first time that in most LGLL subtypes, *STAT3* mutations are associated not only with a more marked neutropenia (as also has been reported by other authors in TCD8^+^-LGLL and CLPD-NK) [7,9,20,30,34], but also with overall reduced counts of major normal residual populations of immune cells in blood, independently of the number of coexisting LGL clones.

Such overall reduced blood counts of (most) normal residual immune-cell subsets may reflect a more profound immunodeficiency status in *STAT3*-mutated LGLL, which is not restricted to neutrophils, but also affects other myeloid cells—i.e., eosinophils, nonclassical monocytes, and dendritic cells—as well as lymphoid cells—i.e., the major T-cell subsets and NK cells. Although the mechanisms responsible for such decreased leukocyte-subset counts in blood of T/NK-LGLL patients remain unknown, the presence of cytopenias (i.e., neutropenia, anemia, and thrombocytopenia) in LGLL, has been associated with an altered production of hematopoietic cells in the BM—similarly to what occurs in children with primary immunodeficiencies (PID) carrying *STAT3* germinal mutations [9,20,35]—and/or an accelerated cell turn-over in the periphery [36,37]. This is supported by the large frequency of BM failure syndromes, such as aplastic anemia, pure red-cell aplasia, hemolytic anemia, paroxysmal nocturnal hemoglobinuria and myelodysplastic syndromes, observed among T-LGLL patients, and to a lesser extend also among CLPD-NK cases [34]. The overall (more pronounced) reduction of blood-cell counts observed for several myeloid and lymphoid populations in *STAT3*-mutated vs. WT LGLL cases might be explained by the fact that common surface molecules shared by all the reduced hematopoietic cells could trigger cytotoxicity by clonal LGLs. In this sense, markers such as CD45, CD244, and HLA-I are present or expressed with higher intensity on the surface of neutrophils, eosinophils, dendritic cells, nonclassical monocytes, and NK cells [38,39,40,41,42] which could be potentially recognized by clonal LGLs, and trigger LGL-mediated cytotoxic mechanisms involved in the death/elimination of the target cells. Further studies are needed to confirm which of these or other markers shared by the different leukocyte-cell subsets decreased in T/NK-LGLL patients are potentially targeted by cytotoxic clonal T/NK-LGLs, particularly in *STAT3*-mutated cases, leading to LGLL-associated cytopenias.

Of note, here we demonstrated absence of *STAT3* mutations in paired (purified) neutrophils from 7/7 *STAT3*-mutated LGLL patients. These results suggest that, in contrast with PID cases that carry *STAT3* mutations, involvement of hematopoietic progenitor cells by *STAT3* mutations is not a frequent finding in LGLL and, thereby, it does not explain the overall decreased production of hematopoietic cells observed in common in both diseases; alternatively, the presence of (over)proliferating *STAT3* mutated LGLs in BM might indirectly impact on normal hematopoiesis, because of resource competition. In contrast, this specific group of PID patients classified as phenocopies of inborn errors of immunity [43,44,45] and characterized by germline *STAT3* gain-of-function (GOF) mutations, also shows systemic autoimmune diseases associated with lymphoproliferation at the expense of clonal T-LGLs. Altogether, these findings would support the existence of underlying autoimmunity mechanisms that lead to the abnormally decreased numbers of many different myeloid- and lymphoid-cell populations reported here and in previous studies in LGLL [1,9,10,46], suggesting the potential existence of shared pathogenic mechanisms for the cytopenias observed in both *STAT3*-mutated PID and LGLL. Thus, most *STAT3* (and also *STAT5B*) mutations detected in both diseases have been identified as GOF mutations that involve the SH2 binding domains of both *STAT* proteins either at dimerization/Sheinermen residues or at the phosphate-binding (pY) and pY+3 peptide binding pocket of the α-helix, BD loop [47]. If this hypothesis holds true, these mutations would lead to upregulation of Th17 cells in parallel to the inhibition of Tregs; this could translate into an increased production of IL-17 and IL-22 that might contribute to the greater rate of autoimmunity and cytopenias reported here for *STAT3*-mutated vs. WT LGLL patients. The presence of (much less pronounced) cytopenias also among the latter LGLL patients in our study could be due to the potential presence in a subset of these patients of *STAT3* mutations outside the SH2 domain, which were not investigated in our cohort. In this regard, recent studies suggest that LGLL patients carrying *STAT3* mutations in the DNA binding domain and the coiled-coil domain of this gene also show a high frequency of neutropenia [25,28,48,49].

Overall, *STAT3* mutations were found in all subtypes of LGLL—but TCD4^+^-LGLL—at percentages that were similar to those previously reported for the major TCD8^+^-LGLL, TCD4^+^-LGLL, and CLPD-NK subtypes [16,17,20,22,23,24,28]. In addition, we further showed the presence of *STAT3* mutations in Tαβ^+^DP- and Tαβ^+^DN-LGLL and almost half of the Tγδ^+^-LGLL cases investigated [18,19,21,24]. In line with the great majority of previous reports, the Y640F mutation was the most frequent *STAT3* mutation (50% in our cohort vs. 28% to 62% in previous studies by others) [16,18,20,22,23,24,25,26,29,30]. In contrast, the *STAT3*-G618R mutation was detected at higher percentages in our clonal LGL populations than in other series (13% vs. 0–7%) [18,24,25,26,29,30]; in turn, the prevalence of other *STAT3* mutations (e.g., S614R, N647I, G656ins, Y657dup, D661V, and D661Y) reported to be rather heterogeneous among the different LGLL categories (0–11%, 0–16%, 0–3%, 0–7%, 0–25%, and 0–72%, respectively) [16,18,20,22,23,24,25,26,29,30], ranged in our cohort from 3% to 7%. Although other mutations at K658 had already been found by others [16,18,20,22,23,29] in T-LGLL, here we first report a case carrying the K658F. Discrepancies in the frequency of distribution of non-*STAT3*-Y640F mutations among the cohorts of LGLL patients reported—including our own study—might be explained by differences in the distribution of the distinct T/NK-LGLL categories in the reported cohorts. As regards *STAT5B* mutations, among the 82 cases with clonal T/NK-LGLs investigated here, only one mutation at the SH2 domain of *STAT5B* was identified (Y665F-mutation, 1% of the whole series) in a TCD4^+^-LGLL patient (1/14 TCD4^+^-LGLL; 7%). Overall the frequency of *STAT5B* mutations in our series is lower than that reported by others, not only among TCD4^+^ (15–55%), but also among TCD8^+^ (0–25%) and Tγδ^+^-LGLL cases (19%), where it has been associated with a worse prognosis [11,20,25,28,30]. In this regard, it should be noted that our patient cohort was not biased by either the clinical condition of the patient or the presence of large expansions of PB LGLs, as all correlative T-LGLL cases referred during the recruitment period were investigated. This might explain the lower frequency of *STAT5B*-mutated cases vs. previous reports, in which more aggressive LGLL cases and/or with larger LGL expansions in PB have been studied.

The presence of the same *STAT3* mutation(s) in different subtypes of T-LGLL and CLPD-NK (e.g., Y640F) further supports the occurrence of similarly (or identical) altered activation pathways across the different subtypes of LGLs, regardless of the specific T/ NK-cell lineage involved. Most intriguing is the presence of different *STAT3* gene mutations in distinct clonal LGL populations from the same patient, which may suggest the existence of an underlying (e.g., genetic or environmental) predisposition for acquisition of *STAT3* mutations. At the same time, it further confirms that *STAT3* mutations in LGL do not occur at early stages of hematopoiesis. These results, together with the coexistence of ≥2 distinct LGL clones in up to one third of TCD8^+^-LGLL cases, support previous studies suggesting that chronic (viral or autologous) antigen stimulation might be involved in the pathogenesis of oligoclonal/clonal LGL expansions and the sequential selection and expansion of a progressively smaller number of LGL clones [10,50,51,52,53]. If this hypothesis holds true, survival of the antigen-selected LGL population(s) might then be maintained via upregulation of specific activation pathways, particularly those involving JAK/STAT signaling [53]. In line with this hypothesis, mutations in several genes other than *STAT3* and *STAT5B* involved in this JAK/STAT signaling pathway, such as the *IGSF3*, *JAK3*, *PTPRT*, *TTN*, and *USH2A* genes, have also been sporadically reported in LGLL [25,27,28,54].

Independently of its biological significance, the demonstration of somatic *STAT3/STAT5B* mutations in LGLs (including coexistence of ≥2—small—LGL populations with different *STAT3* mutations in the same patient) strongly support their clonal nature. Therefore, the presence of *STAT3* mutations would contribute to a more robust demonstration of clonality and diagnosis of T/NK-LGLL, particularly in cases where oligoclonal LGL expansions are observed on phenotypic grounds, and in CLPD-NK patients in whom there are no alternative techniques for confirmation of NK-cell clonality, as shown here. In line with this, it should be noted that during the period of patient recruitment, a total of 10 male patients suspected to have CLPD-NK (by phenotype) were studied; from them, five could not be included in our study, as confirmation of NK-cell clonality could not be done, while the presence of *STAT3* mutations was definitive for NK-cell clonality confirmation in four male (out of 10) patients from our cohort. Of note, in our study *STAT3* and *STAT5B* mutations were investigated using conventional sequencing techniques (i.e., Sanger) on highly-purified clonal T- and NK-LGL fractions, such approach allowing accurate identification of mutations even in cell populations which were present at very low frequency in whole PB and/or corresponded to bi(multi)clonal cases.

At present, the impact of *STAT3* mutations in the clinical behavior and outcome of LGLL remains controversial. Thus, preliminary studies reported an association of *STAT3/STAT5B* mutations with more aggressive disease [18,26,30,31,32]. Overall, our results showed a deleterious impact of *STAT3* mutations in T-LGLL, as reflected by significantly lower platelet counts and a higher frequency of neutropenia (and severe neutropenia), together with a tendency toward a greater prevalence of autoimmune disease conditions, including cytopenias. Altogether, this translated into a significantly higher frequency of *STAT3*-mutated T-LGLL cases requiring therapy (due to the associated-autoimmune disorders). Both T-LGLL and CLPD-NK showed a significantly shorter time-to-therapy in *STAT3*-mutated vs. nonmutated patients, which was more evident in non-Y640F *STAT3*-mutated cases (i.e., G618R, N647I, 656ins, D661V, and D661Y) compared to Y640F-mutated ones; these preliminary results confirm and extend recent reports on the potential impact of the type of *STAT3* mutation on different clinical manifestations of T/NK-LGLL [33]. Despite the low number of cases per LGLL subtype, such more adverse clinical behavior appeared to be shared by distinct subtypes of LGLL, including TCD8^+^-LGLL and Tγδ^+^-LGLL. Additional multicentric studies in larger patient cohorts with longer follow-up are necessary to definitively confirm the prognostic impact of *STAT* mutations in other less frequent LGLL subtypes.

## 4. Materials and Methods

### 4.1. Patients and Samples

One-hundred consecutive patients referred to the Cytometry Service of the University of Salamanca (NUCLEUS) between February 2013 and March 2018 were studied—either at diagnosis (*n* = 79) or during follow-up (*n* = 21)—to confirm/rule out the diagnosis of LGLL. Presence of increased (absolute and/or relative) numbers of LGL cells [10] and/or phenotypically abnormal LGLs in blood (*n* = 87) or bone marrow (BM, *n* = 13) was confirmed in all cases (Table S9): (i) 82 cases had (mono)clonal LGL expansions (66 T-LGLL and 16 CLPD-NK); (ii) six patients showed oligoclonal T-LGL expansions, and (iii) 12 displayed polyclonal LGL lymphocytosis (five T-cell- and seven NK-cell-derived LGL populations). Based on the type of expanded/abnormal T/NK-LGL population(s), patients were further divided into (i) 41/100 (41%) cases that showed expanded TCD8^+^-LGL (33 clonal, four oligoclonal and four polyclonal T-LGL cases); (ii) 23/100 cases (23%) had NK-cell expansions (16 clonal and 7 polyclonal); (iii) 19 (19%) patients displayed expansion of Tγδ^+^ cells (16 clonal, 2 oligoclonal, and 1 polyclonal); (iv) 14 (14%) corresponded to clonal TCD4^+^-LGLL cases; (v) the remaining three cases corresponded to one Tαβ^+^DP and two Tαβ^+^DN clonal cases (Table S9). In-depth analysis of all LGL populations was carried out in 59/82 clonal patients (72%) in whom detailed disease features were available. Of these 59 patients, 45 (76%) showed one-single expanded clone, while expansions of ≥2 different clonal LGL populations were detected in the other 14/59 (24%) cases (Table S9). Median follow-up (from diagnosis) for these patients at the moment of closing the study was of 44 months (range: 1–230 months); 16/68 patients (24%) were treated (with immunosuppressive drugs such as cyclophosphamide, methotrexate, cyclosporine A, and corticoids) either before (*n* = 8) or after (*n* = 8) they were investigated for the presence of *STAT3/STAT5B* mutations (Table 3).

In parallel, 17 patients with T-CLPD other than LGLL (Table S9) and 628 age-matched healthy donors (HD), were analyzed as controls. In addition, polyclonal (normal residual) LGL populations (*n* = 26) and myeloid populations (*n* = 7) from patients with LGL lymphocytosis were also purified and submitted to further mutational analyses (Table S9).

All patients and controls gave their written informed consent to participate in the study, and the study was approved by the Ethics Committee of the University Hospital of Salamanca/IBSAL (Salamanca, Spain).

### 4.2. Immunophenotypic Studies

EDTA-anticoagulated whole blood or BM samples were immunophenotyped using a direct immunofluorescence stain-and-then-lyse technique, based on the EuroFlow T-cell and NK-cell CLPD panels and the EuroFlow standard operating procedures [12,55,56]. Briefly, samples were sequentially stained with the LST tube (“*Lymphocyte Screening Tube*”) for analysis of the distribution of the major leukocyte subsets. Depending on the type of expanded/aberrant cells identified with LST, either the T-cell or the NK-cell CLPD panels were then applied for their further characterization and classification. In T-CLPD, T-cell clonality was assessed prior to staining with the T-CLPD panel using either the IOTest^®^ Beta Mark TCR-Vβ Repertoire Kit (Beckman-Coulter, Brea, CA, USA) in case of Tαβ^+^ T-CLPD or the anti-TCRVγ9 and anti-TCRVδ2 antibody reagents (Beckman-Coulter) in case of Tγδ^+^ T-CLPD (Table S10).

Immediately after sample preparation, stained cells were measured in a FACSCanto-II flow-cytometer (Becton/Dickinson Biosciences, San Jose, CA, USA), using the FACSDiva^TM^ software (Becton/Dickinson Biosciences). Instrument setup, calibration, and daily quality control and monitoring were performed according to well-established EuroFlow protocols [55,56]. For data analysis, the INFINICYT^TM^ software (Cytognos, Salamanca, Spain) was used. The gating strategy employed for the identification of the different T/NK-cell populations, to calculate their relative distribution in blood or BM and to further assess their phenotype, is illustrated in Figures S5 and S6. Absolute cell counts/μL of blood were calculated using a dual platform procedure [57].

### 4.3. Assessment of T- and NK-Cell Clonality on FACS-Sorted Cell Populations 

The clonal nature of the expanded LGL populations was assessed in highly-purified FACS-sorted cells (purified from 3–5 mL of whole PB or BM using a FACSAria-III cytometry—Becton/Dickinson Biosciences, to collect at least 10,000 cells/population with a purity ≥95%), based on the presence of single or a few dominant TCRβ and/or TCRγ VDJ gene rearrangements for T-LGL; for CLPD-NK, the polymerase chain reaction (PCR)-based HUMARA assay for analysis of the pattern of inactivation of the human androgen receptor gene coded in chromosome X of heterozygous female patients was used, following well-established protocols and criteria [14,50,58,59,60]. Those cases in which the sorted LGL population(s) showed one reproducible clonal peak/band by PCR, were considered as “clonal” cases; in turn, those cases in which sorted LGL population(s) with a particular (homogeneous) phenotypic profile showed three or more peaks/bands and a Gaussian curve/smear (with or without minor reproducible peaks/bands), were considered as “oligoclonal” and “polyclonal” cases, respectively [61]. In four CLPD-NK male cases, NK-cell clonality was established on purified cells through confirmation of the presence of *STAT3* (somatic) mutations.

### 4.4. Analysis of STAT3 and STAT5B Gene Mutations

*STAT3* and *STAT5B* gene mutations were analyzed on genomic DNA extracted from all but six highly-purified T/NK-cell populations (159/165 clonal T/NK-LGL populations) from 117 subjects, including (Table S9 and Figure S7): (i) 94/100 clonal LGL populations from 82 subjects; (ii) eight oligoclonal LGL populations from six subjects; (iii) 12 polyclonal LGL populations from 12 subjects; (iv) 19 clonal non-LGL populations from 17 subjects diagnosed with T-CLPD other than T-LGLL; (v) 26 polyclonal T/NK-cell populations (all from clonal LGLL patients). In addition, seven FACS-sorted myeloid-cell populations from seven clonal T-LGLL patients were also analyzed. For these studies, either the GenElute^TM^ Mammalian Genomic DNA Miniprep Kit (Sigma-Aldrich, St Louis, MO, USA) or the Genomic DNA from Tissue Kit (ThermoFisher Scientific, Waltham, MA, USA) were used, as per the recommendations of the manufacturers. Previously reported primers were used for PCR-based amplification of somatic mutational hotspot regions at the *STAT3* SH2 domain at exons 20 and 21 and at the *STAT5B* SH2 domain at exon 16, respectively (Table S11) [11,16,23]. All (forward and reverse) primer pairs produced a single discrete PCR amplicon of the expected length. Amplified products were purified and then sequenced by conventional Sanger techniques at the Genomic Unit of the Cancer Research Center (IBMCC, USAL-CSIC, Salamanca, Spain). All mutations were screened by bidirectional (forward and reverse) sequencing. Sequencing data was analyzed using the Chromas Lite Sequencing Software 2.1.1 (Technelysium, South Brisbane, Australia) and scored as somatic mutations based on their absence in paired non-LGL populations (i.e., normal residual non-LGL TCD4^+^ cells).

### 4.5. Statistical Methods

The nonparametric Mann–Whitney U test (for continuous variables) or the Pearson’s χ2 and Fisher exact tests (for categorical variables), and the log-rank test (for comparison of Kaplan–Meier-based time-to-therapy survival curves) were used for group comparisons, using the IBM-SPSS Statistics software (v25.0, IBM, Armonk, NY, USA). *p*-values ≤0.05 were considered to be associated with statistical significance. To identify phenotypic differences among cell populations from *STAT3* and/or *STAT5B-*mutated vs. *-*unmutated cases and their normal T/NK-cell counterparts, univariate analysis were carried out in a first step, to compare the expression of individual markers between two groups of patients/LGL populations; in a second step, unsupervised (balanced) principal component analysis (PCA) of flow-cytometric data from the whole combinations of markers expressed on >10^6^ individual cells was performed, using the INFINICYT^TM^ software (Cytognos, Salamanca, Spain) [62,63]. Further, unsupervised hierarchical clustering analysis of the overall immunophenotypic profiles for 13 distinct markers (FSC—forward scatter value; SSC—side scatter value; CD2, CD3, CD5, CD8, CD11c, CD16, CD45RA, CD45RO, CD57, HLADR, and Perforin) of *STAT3*-mutated vs. -unmutated TCD8^+^-LGL populations was performed using the gplots R-software package [64] and the resulting data represented in heatmap graphics (Bioinformatics Service, NUCLEUS, USAL).

## 5. Conclusions

Our results show that *STAT3/STAT5B* mutations might be of diagnostic value in LGLL for demonstration of clonality in T-LGLL and CLPD-NK. In addition, we show for the first time that the presence of *STAT3/STAT5B* mutations is associated in LGLL with reduced numbers of most normal residual blood-leukocyte subsets, independently of the specific LGLL cell-lineage involved, with important clinical consequences including a shorter time-to-therapy. Further multicentric studies in large cohorts of LGLL patients with long-term follow-up are needed to confirm these observations.

## Figures and Tables

**Table 1:** Distribution of SH2 domain *STAT3* and *STAT5B* gene mutations in LGLL patients and clonal T/NK-cell populations investigated in this study, grouped per disease category.

**Panel A: Distribution of *STAT3* and *STAT5B* Gene Mutations in the LGLL Patients and Clonal T/NK-Cell Populations**														
**LGL Cell Lineage**					**Mutated Gene**									
					***STAT3*** **Gene Mutations**			***STAT5B*** **Gene Mutations**						**Total**
					**Exon 20**	**Exon 21**		**Exon 16**						
**T-LGLL** Cases 22/66 (33%) *Cell pop.* *25/78 (32%)*		TCD8^+^	N. Cases		1/32 (3%)	11/33 (33%)		0/33 (0%)						12/33 (36%)
			*N. cell pop.*		*3/43 (7%)*	*12/44 (27%)*		*0/44 (0%)*						*15/44 (34%)*
		TCD4^+^	N. Cases		0/14 (0%)	0/14 (0%)		1/14 (7%)						1/14 (7%)
			*N. cell pop.*		*0/15 (0%)*	*0/15 (0%)*		*1/15 (7%)*						*1/15 (7%)*
		Tαβ^+^DP	N. Cases (=*N. cell pop.*)		0/1 (0%)	1/1 (100%)		NA						1/1 (100%)
		Tαβ^+^DN	N. Cases (=*N. cell pop.*)		0/2 (0%)	1/2 (50%)		0/2 (0%)						1/2 (50%)
		Tγδ^+^	N. Cases (=*N. cell pop.*)		2/15 (13%)	5/16 (31%)		0/15 (0%)						7/16 (44%)
**CLPD-NK** 6/16 (38%)		CD56^−/+lo^	N. Cases (=*N. cell pop.*)		0/6 (0%)	4/7 (57%)		0/6 (0%)						4/7 (57%)
		CD56^+^	N. Cases (=*N. cell pop.*)		1/7 (14%)	1/7 (14%)		0/7 (0%)						2/7 (29%)
		CD56^++^	N. Cases (=*N. cell pop.*)		0/2 (0%)	0/2 (0%)		0/2 (0%)						0/2 (0%)
**Total**			N. Cases		4/79 (5%)	23/82 (28%)		1/79 (1%)						28/82 (34%)
			*N. cell pop.*		*6/91 (7%)*	*24/94 (26%)*		*1/91 (1%)*						*31/94 (33%)*
**Panel B: Specific *STAT3/5B* Gene Mutations Identified in Purified Clonal T/NK-cell LGL Populations (*n* = 31) from 28 T/NK-LGLL Patients**														
**Disease Clonal Cell Population Category**			**Mutated Gene**											
			***STAT3*** **Mutations**											***STAT5B*** **Mut**
			**Exon 20**			**Exon 21**								**Exon 16**
			**S614R** **(*n* = 2)**	**G618R** **(*n* = 4)**		**Y640F** **(*n* = 15)**	**N647I** **(*n* = 1)**		**G656ins** **(*n* = 1)**	**Y657dup** **(*n* = 2)**	K658F * **(*n* = 1)**	**D661V** **(*n* = 2)**	**D661Y** **(*n* = 2)**	**Y665F** **(*n* = 1)**
**T-LGLL**	TCD8^+^			3 (20%)		9 (60%)	1 (7%)			1 (7%)			1 (7%)	
	TCD4^+^													1 (100%)
	Tαβ^+^DP					1 (100%)								
	Tαβ^+^DN					1 (100%)								
	Tγδ^+^		2 (29%)			3 (43%)			1 (14%)		1 (14%)			
**CLPD-NK**	CD56^−/+lo^					1 (25%)				1 (25%)		2 (50%)		
	CD56^+^			1 (50%)									1 (50%)	

Results expressed as number (percentage) of mutated LGL cases or cell populations analyzed (in italics) per disease type. Panel B: Empty cell means 0 cases. * Gene mutation not previously described. A total of 3/28 patients showed two different clonal LGL populations with distinct *STAT3* gene mutations: (i) 1/3 cases had the Y640F and Y657dup gene mutations in two different clonal populations of TCD8^+^ cells; (ii) 1/3 cases showed the S614R and G618R *STAT3* gene mutations in one clonal Tγδ^+^ population and one clonal TCD8^+^-cell population, respectively; (iii) 1/3 cases displayed the Y640F and G618R mutations in one clonal CD56^−/+lo^ NK-cell population and one clonal TCD8^+^-cell population, respectively. Abbreviations (alphabetical order): CLPD-NK, chronic lymphoproliferative disorders of NK cells; dup, duplication; LGL, large granular lymphocyte; Mut, mutation; N., number; NA, not analyzed; Pop., populations; SH2, Src homology 2; T-LGLL, leukemia of T large granular lymphocytes.

**Table 2:** Distribution of normal PB leukocyte subsets in T/NK-LGLL according to their *STAT3* or *STAT5B* mutational status.

Cell Populations	Age-Matched HD (*n* = 628)	T/NK-LGLL		*p*-Value
		Wild-Type *STAT3* and *STAT5B* (*n* = 32)	*STAT3* or *STAT5B* Mutated (*n* = 18)	
Granulocytes (/µL)	**3977 (1678–8717)**	**2503 (298–10,264)**	**1308 (46–6676)**	**≤0.008 ^abc^**
Neutrophils	**3709 (1563–8542)**	**2301 (263–10,072)**	**1217 (39–4974)**	**≤0.01 ^abc^**
Eosinophils	**161 (0–876)**	**143 (8.4–1402)**	**30 (1.5–1250)**	**≤0.03 ^abc^**
Basophils	**41 (2.2–222)**	**51 (7.7–161)**	33 (4.3–452)	**0.02 ^a^**; 0.1 ^c^
Monocytes (/µL)	**320 (66–1247)**	**435 (23–1522)**	443 (150–1783)	**0.0001 ^a^**; 0.06 ^b^
cMO	419 (215–1063)	400 (13–1426)	366 (128–1778)	NS
ncMO	**67 (20–166)**	**43 (0–225)**	**40 (0.49–149)**	**≤0.05 ^ab^**
Dendritic cells (/µL)	**24 (4.1–124)**	**17 (0–54)**	**8.2 (0.7–105)**	**≤0.05 ^abc^**
Lymphocytes (/µL)	**1653 (0–5947)**	**2084 (943–7547)**	**1342 (553–4446)**	**0.02 ^ac^**; 0.08 ^b^
T lymphocytes (/µL)	**1188 (371–5298)**	**1672 (655–6469)**	**1109 (291–4039)**	**≤0.05 ^ac^**
TCD8^+^	386 (13–2199)	463 (0–3133)	308 (38–2120)	NS
TCD4^+^	**695 (106–3224)**	**970 (88–3994)**	**686 (227–2221)**	**≤0.05 ^ac^**
Tαβ^+^DP	**8.6 (0–180)**	**28 (2.2–341)**	**24 (3.3–200)**	**≤0.05 ^ab^**
Tαβ^+^DN	15 (2–117)	16 (2.2–94)	13 (5.3–104)	NS
Tγδ^+^	**47 (1–765)**	26 (0–598)	**17 (0–265)**	**0.002 ^b^**; 0.06 ^c^
NK cells (/µL)	**279 (0–1215)**	**150 (2–1383)**	**48 (0–408)**	**≤0.006 ^abc^**
CD56^−/+lo^	5.2 (1.1–19)	2.6 (0–328)	2.6 (0–44)	NS
CD56^+^	**350 (116–773)**	**144 (0–1372)**	**30 (0–403)**	**≤0.002 ^abc^**
CD56^++^	4.5 (1.4–25)	7.8 (1.3–93)	5.5 (0–46)	0.08^a^
B lymphocytes (/µL)	141 (8.1–867)	177 (9.9–970)	147 (15–409)	NS
Plasma cells (/µL)	1.3 (0.104–14)	1.2 (0.091–28)	1.1 (0–14)	NS

Results expressed as median (range) values. In bold: statistically significant differences (*p*-value ≤ 0.05). ^a^ HD vs. WT cases; ^b^ HD vs. *STAT3/5B*-mutated cases; ^c^ *STAT3/5B*-mutated vs. WT cases. Abbreviations (alphabetical order): HD, healthy donor; c, classical; LGLL, leukemia of large granular lymphocytes; MO, monocyte; nc, nonclassical; NS, no statistically significant differences (*p*-value > 0.05); PB, peripheral blood.

**Table 3:** Clinical and biological features of clonal T/NK-LGLL patients (*n* = 82) distributed according to their *STAT3* or *STAT5B* mutational status.

Clinical and Biological Features	T/NK-LGLL		
	*STAT3* and *STAT5B* Wild-Type (*n* = 54)	*STAT3* or *STAT5B* Mutated (*n* = 28)	*p*-Value
Sex (male/female) *	23/31 (43%/57%)	13/15 (46%/54%)	NS
Age (years)	60 ± 18 (4–92)	65 ± 15 (40–90)	NS
Physical examination			
Organomegalies *^1^	5/34 (15%)	9/27 (33%)	NS (0.09)
Skin lesions *	4/33 (12%) ^2^	0/24 (0%)	NS
Peripheral blood cell counts			
Hemoglobin (g/dL)	13 ± 2.1 (8.3–18)	13 ± 2.4 (6.3–17)	NS (0.08)
Platelets (×10^9^/L)	**243 ± 72 (98–383)**	**203 ± 88 (25–421)**	**0.05**
Leukocytes (×10^9^/L)	10 ± 8 (2.7–55)	6.7 ± 6.7 (0.9–36)	NS (0.07)
Clonal LGL cells (×10^9^/L)	4.1 ± 8.9 (0.07–50)	4.1 ± 6.7 (0.5–30)	NS
Low-count clonal LGL lymphocytosis (<0.5 × 10^9^/L) *	14/47 (30%)	6/28 (21%)	NS
Very low-count clonal LGL lymphocytosis (<0.1 × 10^9^/L) *	5/47 (11%)	2/28 (7%)	NS
Cytopenias			
Anemia (≤10 g/dL) *	5/46 (11%)	5/27 (19%)	NS
Thrombocytopenia (≤100 × 10^9^/L) *	1/45 (2%)	3/27 (11%)	NS
Neutropenia (≤1 × 10^9^/L) *	**6/47 (13%)**	**9/28 (32%)**	**0.04**
Severe Neutropenia (≤0.5 × 10^9^/L) *	**0/47 (0%)**	**4/28 (14%)**	**0.02**
Other associated diseases			
Other clonal/neoplastic diseases *	10/32 (31%)	4/25 (16%)	NS
Autoimmune diseases * (including cytopenias)	16/35 (46%)	18/28 (64%)	NS
Autoimmune diseases * (other than cytopenias)	5/33 (15%)	7/28 (25%)	NS
Other diseases *	10/33 (30%)	10/23 (43%)	NS
Outcome and follow-up			
Need for LGLL therapy *^3^	**4/44 (9%)**	**12/24 (50%)**	**0.0001**
Time to LGLL therapy (months) ^#^	**Not reached ^$^**	**72 (1–180)**	**0.0001**
Disease Progression *	2/36 (6%)	3/22 (14%)	NS
Deaths * (overall deaths)	8/44 (18%)	4/24 (17%)	NS
Deaths *^4^	1/44 (2%)	2/23 (9%)	NS

Results expressed as mean ± standard deviation (SD) (and range), * as number of cases (percentage) or ^#^ as median (95% confidence interval). In bold: statistically significant differences (*p*-value ≤ 0.05). ^$^ After a median follow at of 183. ^1^ Adenopathy, splenomegaly, and/or hepatomegaly. ^2^ Cutaneous lesions corresponding to skin lesions of a coexisting mastocytosis, scleroderma, oro-buccal lesions, and cutaneous follicular NHL. ^3^ In all cases, treatment was administered because of the presence of cytopenias and/or other associated autoimmune diseases: WT cases were treated with cyclophosphamide (*n* = 2), corticoids (*n* = 1), and corticoids plus methotrexate (*n* = 1); *STAT3* mutated cases were treated with cyclophosphamide (*n* = 1), methotrexate (*n* = 2), corticoids (*n* = 2), cyclosporine A (*n* = 2), and combinations of any of the above therapeutic agents plus corticoids and/or hematopoietic colony stimulating factors (*n* = 5). ^4^ Complications derived from the LGLL-associated autoimmune disease. Abbreviations (alphabetical order): LGLL, large granular lymphocytic leukemia; NS, no statistically significant differences (*p*-value > 0.05).

## Supplementary Materials

The following are available online at https://www.mdpi.com/2072-6694/12/12/3508/s1, Figure S1: Lollipop diagrams representing the specific *STAT3* (A) and *STAT5B* (B) mutations identified in our T/NK-LGLL cohort and their relative position in the SH2 domain of both genes, Figure S2: Multidimensional phenotypic comparisons between wild-type and *STAT3* or *STAT5B* mutated populations of clonal LGLL cells, Figure S3: Distribution of clonal and normal residual peripheral blood cells in TCD8^+^-LGLL patients classified according to their *STAT3* mutational status and the presence of biclonal vs. monoclonal LGL, Figure S4: Distribution of distinct maturation-associated cell subsets for the major normal residual T cells in WT vs. *STAT3*-mutated vs. age-matched HD in TCD8^+^- and Tγδ^+^-LGLL, Figure S5: Phenotypic identification of distinct subtypes of aberrant clonal LGL of different T-cell lineages in T-LGLL patients, Figure S6: Phenotypic identification of distinct subtypes of aberrant clonal NK-cells in CLPD-NK patients, Figure S7: Flow-chart illustrating how the cases and cell populations were selected for the analysis of *STAT3* and *STAT5B* mutations, Table S1: Distribution of *STAT3* or *STAT5B-*mutated T-LGL cell populations according to the T-cell receptor beta chain variable region (TCR-Vβ) or T-cell receptor gamma 9 and delta 2 chain region (TCR-Vγ9 and TCR-Vδ2) expressed, Table S2: Distribution of normal PB leukocyte subsets in T-LGLL according to their *STAT3* or *STAT5B* mutational status, Table S3: Distribution of normal PB leukocyte subsets in TCD8^+^-LGLL according to their *STAT3* mutational status, Table S4: Distribution of normal PB leukocyte subsets in Tγδ^+^-LGLL according to their *STAT3* mutational status, Table S5: Distribution of normal PB leukocyte subsets in CLPD-NK according to their STAT3 mutational status, Table S6: Clinical and biological features of clonal T-LGLL cases with wild-type (WT) vs. mutated *STAT3/5B*, Table S7: Clinical and biological features of TCD8^+^-LGLL according to their *STAT3* mutational status and the presence of bi(multi) vs. monoclonal LGL populations, Table S8: Clinical and biological features of clonal CLPD-NK cases with wild-type (WT) vs. mutated *STAT3*, Table S9: Distribution of LGLL cases and non-LGL CLPD patients (*n* = 117) included in this study and the corresponding populations of LGL identified (*n* = 165) classified according to their phenotypic profile and (mono vs. oligo/poly)clonal nature, Table S10: Panels of fluorochrome-conjugated antibodies (specificities and sources) used for sequential immunophenotypic analyses of LGLL by flow cytometry, Table S11: *STAT3* and *STAT5B* primers and temperature conditions used for PCR-based gene mutation analysis.

## References

[1] Loughran T.P., Kadin M.E., Starkebaum G., Abkowitz J.L., Clark E.A., Disteche C., Lum L.G., Slichter S.J. (1985). Leukemia of large granular lymphocytes: Association with clonal chromosomal abnormalities and autoimmune neutropenia, thrombocytopenia, and hemolytic anemia. Ann. Intern. Med..

[2] Semenzato G., Zambello R., Starkebaum G., Oshimi K., Loughran T.P. (1997). The lymphoproliferative disease of granular lymphocytes: Updated criteria for diagnosis. Blood.

[3] Loughran T.P. (1993). Clonal diseases of large granular lymphocytes. Blood.

[4] Swerdlow S.H., Campo E., Harris N.L., Jaffe E.S., Pileri S.A., Stein H., Thiele J. (2017). WHO Classification of Tumours of Haematopoietic and Lymphoid Tissues.

[5] Zambello R., Teramo A., Gattazzo C., Semenzato G. (2014). Are T-LGL Leukemia and NK-Chronic Lymphoproliferative Disorder really two distinct diseases?. Transl. Med.^®^ UniSa.

[6] Clemente M.J., Wlodarski M.W., Makishima H., Viny A.D., Bretschneider I., Shaik M., Bejanyan N., Lichtin A.E., His E.D., Paquette R.L. (2011). Clonal drift demonstrates unexpected dynamics of the T-cell repertoire in T-large granular lymphocyte leukemia. Blood.

[7] Poullot E., Zambello R., Leblanc F., Bareau B., De March E., Roussel M., Boulland M.L., Houot R., Renault A., Fest T. (2014). Chronic natural killer lymphoproliferative disorders: Characteristics of an international cohort of 70 patients. Ann. Oncol..

[8] Lima M., Almeida J., Dos Anjos Teixeira M., Del Carmen Alguero M., Santos A.H., Balanzategui A., Queirós M.L., Bárcena P., Izarra A., Fonseca S. (2003). TCRαβ+/CD4+ large granular lymphocytosis: A new clonal T-cell lymphoproliferative disorder. Am. J. Pathol..

[9] Oshimi K. (2017). Clinical Features, Pathogenesis, and Treatment of Large Granular Lymphocyte Leukemias. Intern. Med..

[10] Lamy T., Moignet A., Loughran T.P. (2017). LGL leukemia: From pathogenesis to treatment. Blood.

[11] Rajala H.L.M., Eldfors S., Kuusanmäki H., Van Adrichem A.J., Olson T., Lagström S., Andersson E.I., Jerez A., Clemente M.J., Yan Y. (2013). Discovery of somatic STAT5b mutations in large granular lymphocytic leukemia. Blood.

[12] Van Dongen J.J., Lhermitte L., Böttcher S., Almeida J., van der Velden V.H., Flores-Montero J., Rawstron A., Asnafi V., Lécrevisse Q., Lucio P. (2012). EuroFlow antibody panels for standardized n-dimensional flow cytometric immunophenotyping of normal, reactive and malignant leukocytes. Leukemia.

[13] Matutes E. (2017). Large granular lymphocytic leukemia. Current diagnostic and therapeutic approaches and novel treatment options. Expert Rev. Hematol..

[14] Bárcena P., Jara-Acevedo M., Tabernero M.D., López A., Sánchez M.L., García-Montero A.C., Muñoz-García N., Vidriales M.B., Paiva A., Lecrevisse Q. (2015). Phenotypic profile of expanded NK cells in chronic lymphoproliferative disorders: A surrogate marker for NK-cell clonality. Oncotarget.

[15] Ohgami R.S., Ohgami J.K., Pereira I.T., Gitana G., Zehnder J.L., Arber D.A. (2011). Refining the diagnosis of T-cell large granular lymphocytic leukemia by combining distinct patterns of antigen expression with T-cell clonality studies. Leukemia.

[16] Koskela H.L.M., Eldfors S., Ellonen P., Van Adrichem A.J., Kuusanmäki H., Andersson E.I., Lagström S., Clemente M.J., Olson T., Jalkanen S.E. (2012). Somatic STAT3 mutations in Large Granular Lymphocytic Leukemia. N. Engl. J. Med..

[17] Rajala H.L.M., Eldfors S., Kuusanmaki H., Andersson E.I., van Adrichem A.J., Lagstrom S., Olson T., Jerez A., Clemente M.J., Zhang D. (2012). Discovery of STAT5b Mutations and Small Subclones of STAT3 Mutations in Large Granular Lymphocytic (LGL) Leukemia. Blood.

[18] Jerez A., Clemente M.J., Makishima H., Koskela H., Leblanc F., Ng K.P., Olson T., Przychodzen B., Afable M., Gomez-Segui I. (2012). STAT3 mutations unify the pathogenesis of chronic lymphoproliferative disorders of NK cells and T cell large granular lymphocyte leukemia. Blood.

[19] Shi M., He R., Feldman A.L., Viswanatha D.S., Jevremovic D., Chen D., Morice W.G. (2018). *STAT3* mutation and its clinical and histopathologic correlation in T-cell large granular lymphocytic leukemia. Hum. Pathol..

[20] Teramo A., Barilà G., Calabretto G., Ercolin C., Lamy T., Moignet A., Roussel M., Pastoret C., Leoncin M., Gattazzo C. (2017). *STAT3* mutation impacts biological and clinical features of T-LGL leukemia. Oncotarget.

[21] Tanahashi T., Sekiguchi N., Matsuda K., Takezawa Y., Ito T., Kobayashi H., Ichikawa N., Nishina S., Senoo N., Sakai H. (2016). Cell size variations of large granular lymphocyte leukemia: Implication of a small cell subtype of granular lymphocyte leukemia with STAT3 mutations. Leuk. Res..

[22] Rajala H.L.M., Olson T., Clemente M.J., Lagström S., Ellonen P., Lundan T., Hamm D.E., Arshi Uz Zaman S., Lopez Marti J.M., Andersson E.I. (2015). The analysis of clonal diversity and therapy responses using STAT3 mutations as a molecular marker in large granular lymphocytic leukemia. Haematologica.

[23] Fasan A., Kern W., Grossmann V., Haferlach C., Haferlach T., Schnittger S. (2013). STAT3 mutations are highly specific for large granular lymphocytic leukemia. Leukemia.

[24] Ishida F., Matsuda K., Sekiguchi N., Makishima H., Taira C., Momose K., Nishina S., Senoo N., Sakai H., Ito T. (2014). *STAT3* gene mutations and their association with pure red cell aplasia in large granular lymphocyte leukemia. Cancer Sci..

[25] Coppe A., Andersson E.I., Binatti A., Gasparini V.R., Bortoluzzi S., Clemente M., Herling M., Maciejewski J., Mustjoki S., Bortoluzzi S. (2017). Genomic landscape characterization of large granular lymphocyte leukemia with a systems genetics approach. Leukemia.

[26] Qiu Z., Fan L., Wang R., Gale R.P., Liang H., Wang L., Wu Y., Qiao C., Chen Y., Xu W. (2016). Methotrexate therapy of T-cell large granular lymphocytic leukemia impact of *STAT3* mutation. Oncotarget.

[27] Kurt H., Jorgensen J.L., Amin H.M., Patel K.P., Wang S.A., Lin P., Kanagal-Shamanna R., Loghavi S., Thakral B., Khogeer H.A. (2018). Chronic lymphoproliferative disorder of NK-cells: A single-institution review with emphasis on relative utility of multimodality diagnostic tools. Eur. J. Haematol..

[28] Andersson E.I., Tanahashi T., Sekiguchi N., Gasparini V.R., Bortoluzzi S., Kawakami T., Matsuda K., Mitsui T., Eldfors S., Bortoluzzi S. (2016). High incidence of activating *STAT5B* mutations in CD4-positive T-cell large granular lymphocyte leukemia. Blood.

[29] Kristensen T., Larsen M., Rewes A., Frederiksen H., Thomassen M., Møller M.B. (2014). Clinical relevance of sensitive and quantitative STAT3 mutation analysis using next-generation sequencing in T-cell large granular lymphocytic leukemia. J. Mol. Diagn..

[30] Barilà G., Teramo A., Calabretto G., Vicenzetto C., Gasparini V.R., Pavan L., Leoncin M., Vedovato S., Frigo A.C., Facco M. (2020). Stat3 mutations impact on overall survival in large granular lymphocyte leukemia: A single-center experience of 205 patients. Leukemia.

[31] Sanikommu S.R., Clemente M.J., Chomczynski P., Afable M.G., Jerez A., Thota S., Patel B., Hirsch C., Aziz Nazha J.D., Lichtin A. (2018). Clinical features and treatment outcomes in large granular lymphocytic leukemia (LGLL). Leuk. Lymphoma.

[32] Zhu Y., Gao Q., Hu J., Liu X., Guan D., Zhang F. (2020). Clinical features and treatment outcomes in patients with T-cell large granular lymphocytic leukemia: A single-institution experience. Leuk. Res..

[33] Olson K.C., Moosic K.B., Jones M.K., Larkin P.M.K., Olson T.L., Toro M.F., Fox T.E., Feith D.J., Loughran T.P. (2020). Large granular lymphocyte leukemia serum and corresponding hematological parameters reveal unique cytokine and sphingolipid biomarkers and associations with STAT3 mutations. Cancer Med..

[34] Sun H., Wei S., Yang L. (2019). Dysfunction of immune system in the development of large granular lymphocyte leukemia. Hematology.

[35] Zhang R., Shah M.V., Loughran T.P. (2010). The root of many evils: Indolent large granular lymphocyte leukaemia and associated disorders. Hematol. Oncol..

[36] Bockorny B., Dasanu C.A. (2012). Autoimmune manifestations in large granular lymphocyte leukemia. Clin. Lymphoma Myeloma Leuk..

[37] Liu J.H., Wei S., Lamy T., Epling-Burnette P.K., Starkebaum G., Djeu J.Y., Loughran T.P. (2000). Chronic neutropenia mediated by Fas ligand. Blood.

[38] Assarsson E., Kambayashi T., Schatzle J.D., Cramer S.O., von Bonin A., Jensen P.E., Ljunggren H.-G., Chambers B.J. (2004). NK Cells Stimulate Proliferation of T and NK Cells through 2B4/CD48 Interactions. J. Immunol..

[39] Pahima H., Puzzovio P.G., Levi-Schaffer F. (2019). 2B4 and CD48: A powerful couple of the immune system. Clin. Immunol..

[40] McArdel S.L., Terhorstb C., Sharpe A.H. (2016). Roles of CD48 in regulating immunity and tolerance. Clin. Immunol..

[41] Bernson E., Christenson K., Pesce S., Pasanen M., Marcenaro E., Sivori S., Thorén F.B. (2019). Downregulation of HLA Class I Renders Inflammatory Neutrophils More Susceptible to NK Cell-Induced Apoptosis. Front. Immunol..

[42] Angénieux C., Dupuis A., Gachet C., de la Salle H., Maître B. (2019). Cell surface expression of HLA I molecules as a marker of young platelets. J. Thromb. Haemost..

[43] Picard C., Gaspar H.B., Al-herz W., Bousfiha A., Sullivan K.E. (2018). International Union of Immunological Societies: 2017 Primary Immunodeficiency Diseases Committee Report on Inborn Errors of Immunity. J. Clin. Immunol..

[44] Consonni F., Dotta L., Todaro F., Vairo D., Badolato R. (2017). Signal transducer and activator of transcription gain-of-function primary immunodeficiency/immunodysregulation disorders. Curr. Opin. Pediatr..

[45] Lorenzini T., Dotta L., Giacomelli M., Vairo D., Badolato R. (2017). STAT mutations as program switchers: Turning primary immunodeficiencies into autoimmune diseases. J. Leukoc. Biol..

[46] Schultz K.R. (2013). STAT3 mutations and persistence of autoimmunity. Blood.

[47] De Araujo E.D., Orlova A., Neubauer H.A., Bajusz D., Seo H., Dhe-paganon S., Keser G.M., Moriggl R., Gunning P.T. (2019). Structural Implications of STAT3 and STAT5 SH2 Domain Mutations. Cancers.

[48] Andersson E., Kuusanmäki H., Bortoluzzi S., Lagström S., Parsons A., Rajala H., van Adrichem A., Eldfors S., Olson T., Clemente M.J. (2016). Activating somatic mutations outside the SH2-domain of STAT3 in LGL-Leukemia. Leukemia.

[49] Shahmarvand N., Nagy A., Shahryari J., Ohgami R.S. (2018). Mutations in the signal transducer and activator of transcription family of genes in cancer. Cancer Sci..

[50] Sandberg Y., Almeida J., Gonzalez M., Lima M., Bárcena P., Szczepañski T., van Gastel-Mol E., Wind H., Balanzategui A., van Dongen J. (2006). TCRgd+ large granular lymphocyte leukemias reflect the spectrum of normal antigen-selected TCRgd+ T-cells. Leukemia.

[51] Singleton T.P., Yin B., Teferra A., Mao J.Z. (2015). Spectrum of Clonal Large Granular Lymphocytes (LGLs) of αβ T Cells T-Cell Clones of Undetermined Significance, T-Cell LGL Leukemias, and T-Cell Immunoclones. Am. J. Clin. Pathol..

[52] Lima M., Almeida J., Santos A.H., Teixeira A., Alguero C., Luı M., Gonzalez M., Miguel F.S., Orfa A. (2001). Immunophenotypic Analysis of the TCR-Vβ Repertoire in 98 Persistent Expansions of CD3+/TCR-αβ+ Large Granular Lymphocytes Utility in Assessing Clonality and Insights into the Pathogenesis of the Disease. Am. J. Pathol..

[53] Teramo A., Barilà G., Calabretto G., Vicenzetto C., Gasparini V.R., Semenzato G., Zambello R. (2020). Insights Into Genetic Landscape of Large Granular Lymphocyte Leukemia. Front. Oncol..

[54] Andersson E.I., Rajala H.L.M., Eldfors S., Ellonen P., Olson T., Jerez A., Clemente M.J., Kallioniemi O., Porkka K., Heckman C. (2013). Novel somatic mutations in large granular lymphocytic leukemia affecting the STAT-pathway and T-cell activation. Blood Cancer J..

[55] Kalina T., Flores-Montero J., van der Velden V.H.J., Martin-Ayuso M., Böttcher S., Ritgen M., Almeida J., Lhermitte L., Asnafi V., Mendonça A. (2012). EuroFlow standardization of flow cytometer instrument settings and immunophenotyping protocols. Leukemia.

[56] EuroFlow. https://www.euroflow.org/.

[57] Hultin L.E., Chow M., Jamieson B.D., O’Gorman M.R.G., Menendez F.A., Borowski L., Denny T.N., Margolick J.B. (2010). Comparison of interlaboratory variation in absolute T-cell counts by single-platform and optimized dual-platform methods. Cytom. B Clin. Cytom..

[58] Langerak A.W., Den Beemd V., Wolvers-tettero I.L.M., Boor P.P.C., Van Lochem E.G., Hooijkaas H., Van Dongen J.J.M. (2001). Molecular and flow cytometric analysis of the VB repertoire for clonality assessment in mature TCRab T-cell proliferations. Blood.

[59] Van Dongen J.J.M., Langerak A.W., Brüggemann M., Evans P.A.S., Hummel M., Lavender F.L., Delabesse E., Davi F., Schuuring E., García-Sanz R. (2003). Design and standardization of PCR primers and protocols for detection of clonal immunoglobulin and T-cell receptor gene recombinations in suspect lymphoproliferations: Report of the BIOMED-2 concerted action BMH4-CT98-3936. Leukemia.

[60] Kopp P., Jaggi R., Tobler A., Borisch B., Oestreicher M., Sabacan L., Jameson J.L., Fey M.F. (1997). Clonal X-inactivation analysis of human tumours using the human androgen receptor gene (HUMARA) polymorphism: A non-radioactive and semiquantitative strategy applicable to fresh and archival tissue. Mol. Cell. Probes.

[61] Langerak A.W., Groenen P.J.T.A., Brüggemann M., Beldjord K., Bellan C., Bonello L., Boone E., Carter G.I., Catherwood M., Davi F. (2012). EuroClonality/BIOMED-2 guidelines for interpretation and reporting of Ig/TCR clonality testing in suspected lymphoproliferations. Leukemia.

[62] Pedreira C.E., Costa E.S., Barrena S., Lecrevisse Q., Almeida J., Van Dongen J.J.M., Orfao A. (2008). Generation of flow cytometry data files with a potentially infinite number of dimensions. Cytom. A.

[63] Costa E.S., Pedreira C.E., Barrena S., Lecrevisse Q., Flores J., Quijano S., Almeida J., García-MacIas M.C., Bottcher S., Van Dongen J.J.M. (2010). Automated pattern-guided principal component analysis vs expert-based immunophenotypic classification of B-cell chronic lymphoproliferative disorders: A step forward in the standardization of clinical immunophenotyping. Leukemia.

[64] gplots: Various R Programming Tools for Plotting Data. https://cran.r-project.org/web/packages/gplots/index.html.

