# Supplementary material for: STAT3 and STAT5B Mutations in T/NK-Cell Chronic Lymphoproliferative Disorders of Large Granular Lymphocytes (LGL): Association with Disease Features
Source: Cancers (Basel). 2020 Nov 25;12(12):3508. doi: 10.3390/cancers12123508 (PMC7760806; doi:10.3390/cancers12123508)
Supplement: Supplementary file 1 [file cancers-12-03508-s001.pdf]

cancers

MDPI

Supplementary Materials: *STAT3* and *STAT5B* Mutations in T/NK–Cell Chronic Lymphoproliferative Disorders of Large Granular Lymphocytes (LGL): Association with Disease Features

Noemí Muñoz–García, María Jara–Acevedo, Carolina Caldas, Paloma Bárcena, Antonio López, Noemí Puig, Miguel Alcoceba, Paula Fernández, Neus Villamor, Juan A. Flores–Montero, Karoll Gómez, María Angelina Lemes, Jose Carlos Hernández, Iván Álvarez–Twose, Jose Luis Guerra, Marcos González, Alberto Orfao and Julia Almeida, on behalf of the EuroFlow Consortium

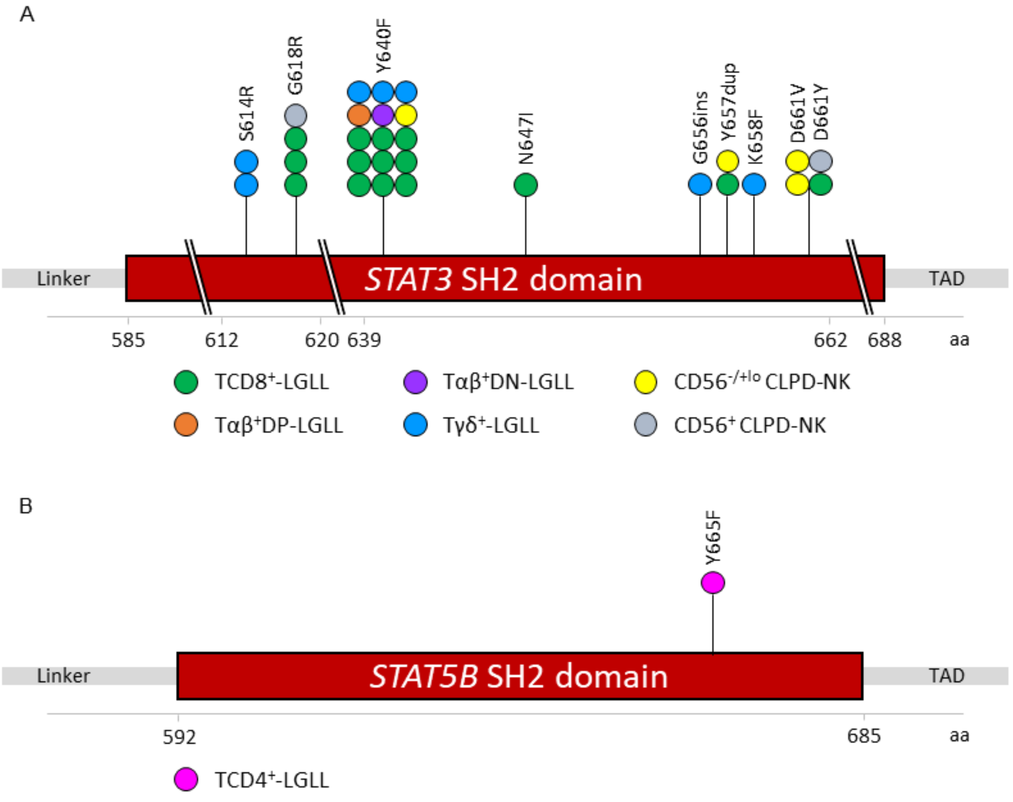

Figure S1. Lollipop diagrams representing the specific *STAT3* (**A**) and *STAT5B* (**B**) mutations identified in our T/NK–LGLL cohort and their relative position in the SH2 domain of both genes. Each circle represents one clonal T/NK–LGL population. Abbreviations (alphabetical order): aa, amino acid; LGLL, leukemia of large granular lymphocytes; SH2, Src homology 2; TAD, transactivation domain.

A. Tγδ^{+}-LGLL cell populations

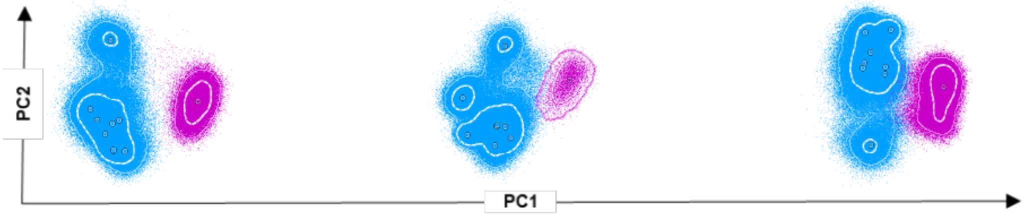

Parameter (PC1)	Significance (%)	Expression
CD11c	16	↑
HLADR	11	↑
CD57	10	-

Parameter (PC1)	Significance (%)	Expression
CD57	16	-
CyGranzyme B	14	↓
CD279	11	↑

Parameter (PC1)	Significance (%)	Expression
CD11c	11	↑
CD57	10	-
CD8	10	↑

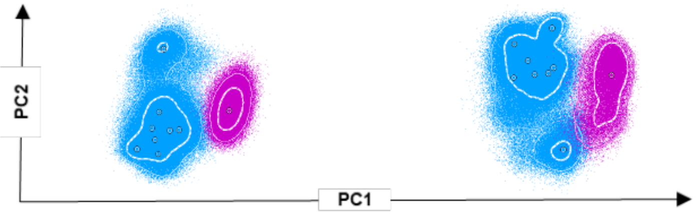

Parameter (PC1)	Significance (%)	Expression
CD11c	16	↑
CD94	13	-
CD7	12	↓
CD5	12	↓

Parameter (PC1)	Significance (%)	Expression
CD8	14	↑
CD57	13	-
CD11c	11	↑
CD45RA	10	↑

B. CD56$^{-/+lo}$ CLPD-NK cell populations

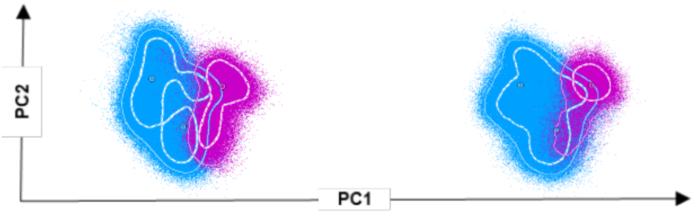

Parameter (PC1)	Significance (%)	Expression
CD16	16	↑
CD11c	13	↓
CD7	13	↑
CyGranzyme B	11	↑

Parameter (PC1)	Significance (%)	Expression
CD57	19	↓
HLADR	17	↑
CD7	16	↑
FSC-A	13	↑

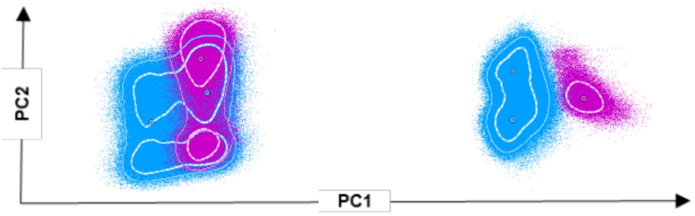

Parameter (PC1)	Significance (%)	Expression
HLADR	22	-
CD94	18	-
CD7	16	-

Parameter (PC1)	Significance (%)	Expression
CyPerforin	15	↓
CD57	15	↓
CD11c	12	↓
CD7	11	↓
CD26	11	↑
HLADR	11	↑

C. TCD4⁺-LGLL cell populations

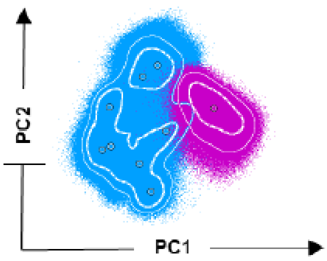

Parameter (PC1)	Significance (%)	Expression
CD45RO	27	↓
CD2	12	↓
CyPerforin	8.8	↑
CD94	7.7	↑
CD16	6.3	↑
CD11c	6.0	↑

CD57 and CyGranzyme B not valuable (excluded from the analysis)

D. Tαβ⁺DN-LGLL cell populations

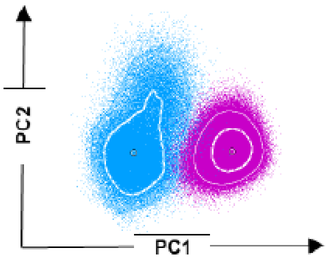

Parameter (PC1)	Significance (%)	Expression
CD16	16	↓
CD8	13	↓
CD45RA	12	↑
HLADR	9.2	↑
CD11c	8.6	↓
CD94	6.6	↓

CD57 not valuable (excluded from the analysis)

E. CD56⁺ CLPD-NK cell populations

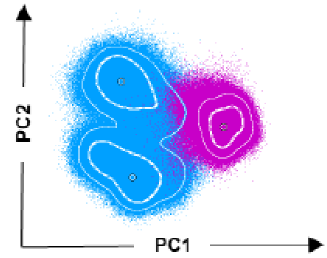

Parameter (PC1)	Significance (%)	Expression
CD57	20	↓
CD11c	20	↑
CD7	18	↓
CD56	13	↓
CD26	6.5	↓
CyGranzyme B	5.4	↑

Figure S2. Multidimensional phenotypic comparisons between wild–type and *STAT3* or *STAT5B* mutated populations of clonal LGLL cells. Comparison between each *STAT3* or *STAT5B* mutated case (in purple) vs. all non–mutated cell population(s) (coloured blue). Overexpression (↑), underexpression (↓) or non–differential expression (–) of markers in mutated vs. wild–type cell populations. In all APS1 (automatic population separator 1) graphical representations, solid circles represent median values for all phenotypic parameters evaluated, and the inner (dotted) and outer (solid) lines represent the first and the second standard deviation for each population identified.

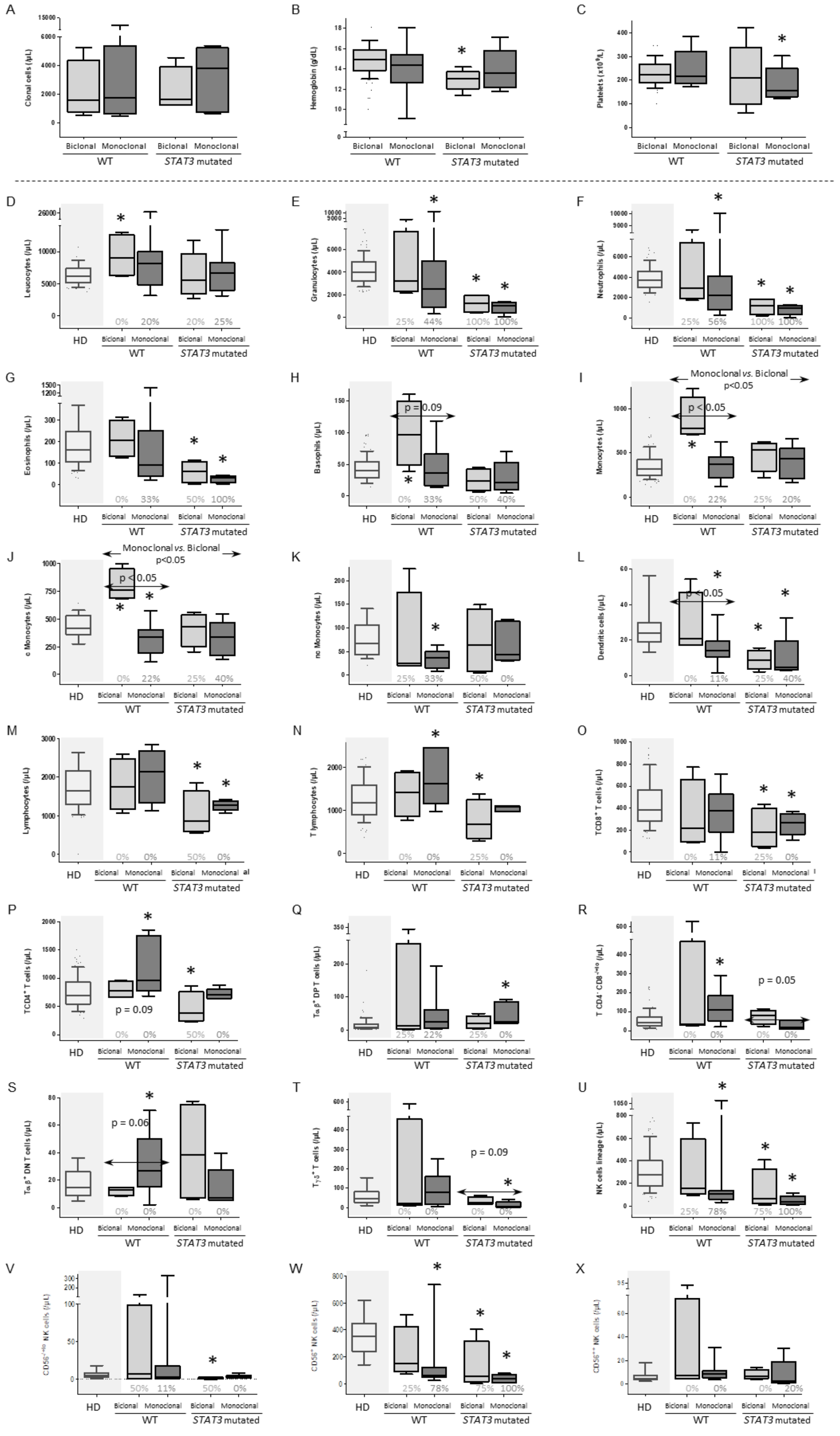
A
B
C
D
E
F
G
H
I
J
K
L
M
N
O
P
Q
R
S
T
U
V
W
X
Clonal cells (/µL)
Hemoglobin (g/dL)
Platelets (x10⁹/L)
Leucocytes (/µL)
Granulocytes (/µL)
Neutrophils (/µL)
Eosinophils (/µL)
Basophils (/µL)
Monocytes (/µL)
c Monocytes (/µL)
nc Monocytes (/µL)
Dendritic cells (/µL)
Lymphocytes (/µL)
T lymphocytes (/µL)
Monoclonal vs. Biclonal p<0.05
p = 0.09
p < 0.05
p = 0.05
p = 0.06
HD
Biclonal
Monoclonal
WT
STAT3 mutated

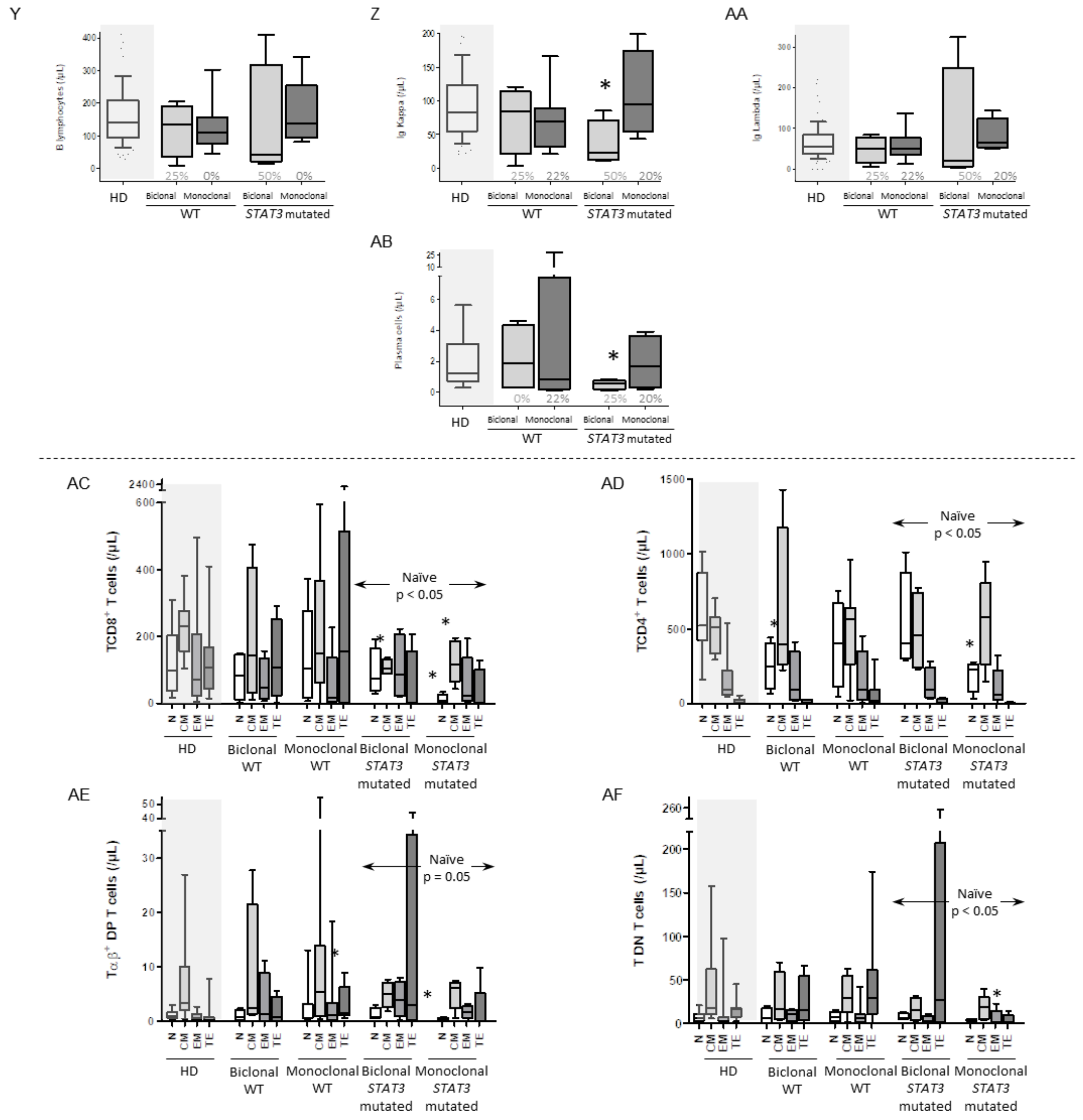

Figure S3. Distribution of clonal and normal residual peripheral blood cells in TCD8+–LGLL patients classified according to their *STAT3* mutational status and the presence of biclonal vs. monoclonal LGL. (**Panels A–C**): Number of clonal LGL in blood, as well as hemoglobin levels and platelet counts observed at diagnosis in TCD8+–LGLL patients according to their *STAT3* mutational status and the presence of bi(multi) vs. monoclonality. (**Panels D–AB**): Distribution of normal leukocyte subsets in TCD8+–LGLL patients according to their *STAT3* mutational status and the presence of bi(multi) vs. monoclonality. (**Panels AC–AF**): Distribution of normal residual T–cells at different maturational stages in TCD8+–LGLL patients classified according to their *STAT3* mutational status and the presence of bi(multi) vs. monoclonality. Distribution of clonal and normal residual peripheral blood cell counts in bi(multi)clonal WT (n = 4), monoclonal WT (n = 9), bi(multi)clonal *STAT3*–mutated (n = 4) and monoclonal *STAT3*–mutated cases (n = 5) with at least one expanded clone of TCD8+–LGL cells vs. aged–matched HD (n = 638, including 628 for comparisons of the distribution of residual normal immune cells and 10 for comparisons of the maturation–associated cell subsets of major normal residual T–cell populations). Notched boxes represent 25th and 75th percentile values; the lines in the middle correspond to median values (50th percentile) and vertical lines represent 10th and 90th percentile values. The number under the boxes means the percentage of cases that have lower values than the minimum value observed among HD. *p–value ≤ 0.05 vs. HD. Abbreviations (alphabetical order): C, classical; CM, central memory; EM, effector memory; HD, healthy donors; LGL, large granular lymphocytes; LGLL, leukemia of LGL; N, naïve; NC, non–classical; p, p–value; TE, terminal effector; WT, wild–type.

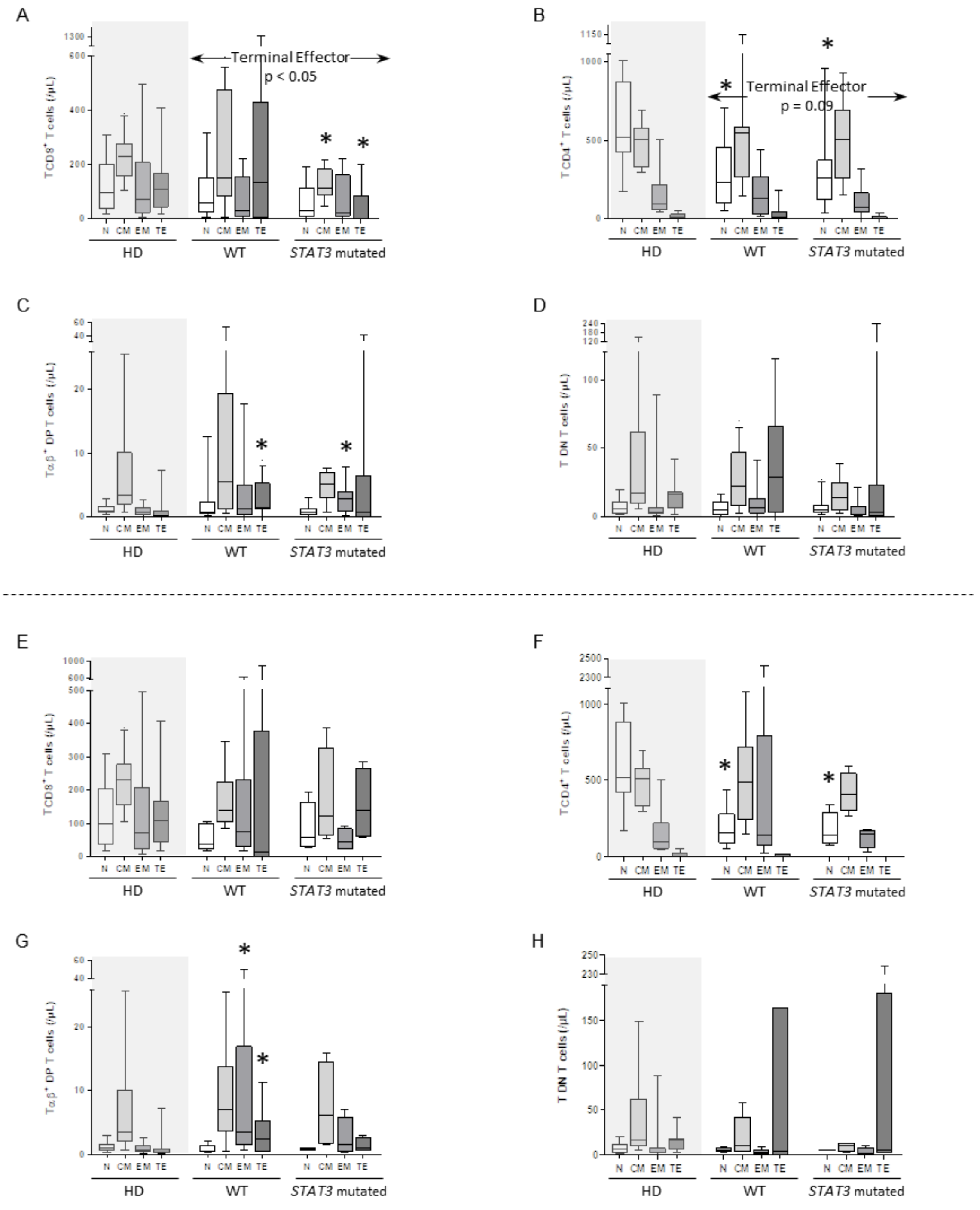

Figure S4. Distribution of distinct maturation–associated cell subsets for the major normal residual T cells in WT vs. *STAT3*–mutated vs. age–matched HD in TCD8+– and Tγδ+–LGLL. (**Panels A–D**): Distribution of normal residual T cells at different maturational stages in TCD8+–LGLL patients classified according to their *STAT3* mutational status. (**Panels E–H**): Distribution of normal residual T–cells at different maturational stages in Tγδ+–LGLL patients classified according to their *STAT3* mutational status. Distribution of clonal and normal residual peripheral blood cell counts in WT (*n* = 15 and *n* = 6) and *STAT3* mutated (*n* = 10 and *n* = 4) vs. aged–matched HD (*n* = 10) for TCD8+–LGLL and Tγδ+–LGLL patients. Notched boxes represent 25th and 75th percentile values; the lines in the middle correspond to median values (50th percentile) and vertical lines represent 10th and 90th percentile values. *p–value ≤ 0.05 vs. HD. Abbreviations (alphabetical order): CM, central memory; EM, effector memory; HD, healthy donors; LGL, large granular lymphocytes; LGLL, leukemia of LGL; N, naïve; *p*, *p*–value; TE, terminal effector; WT, wild–type.

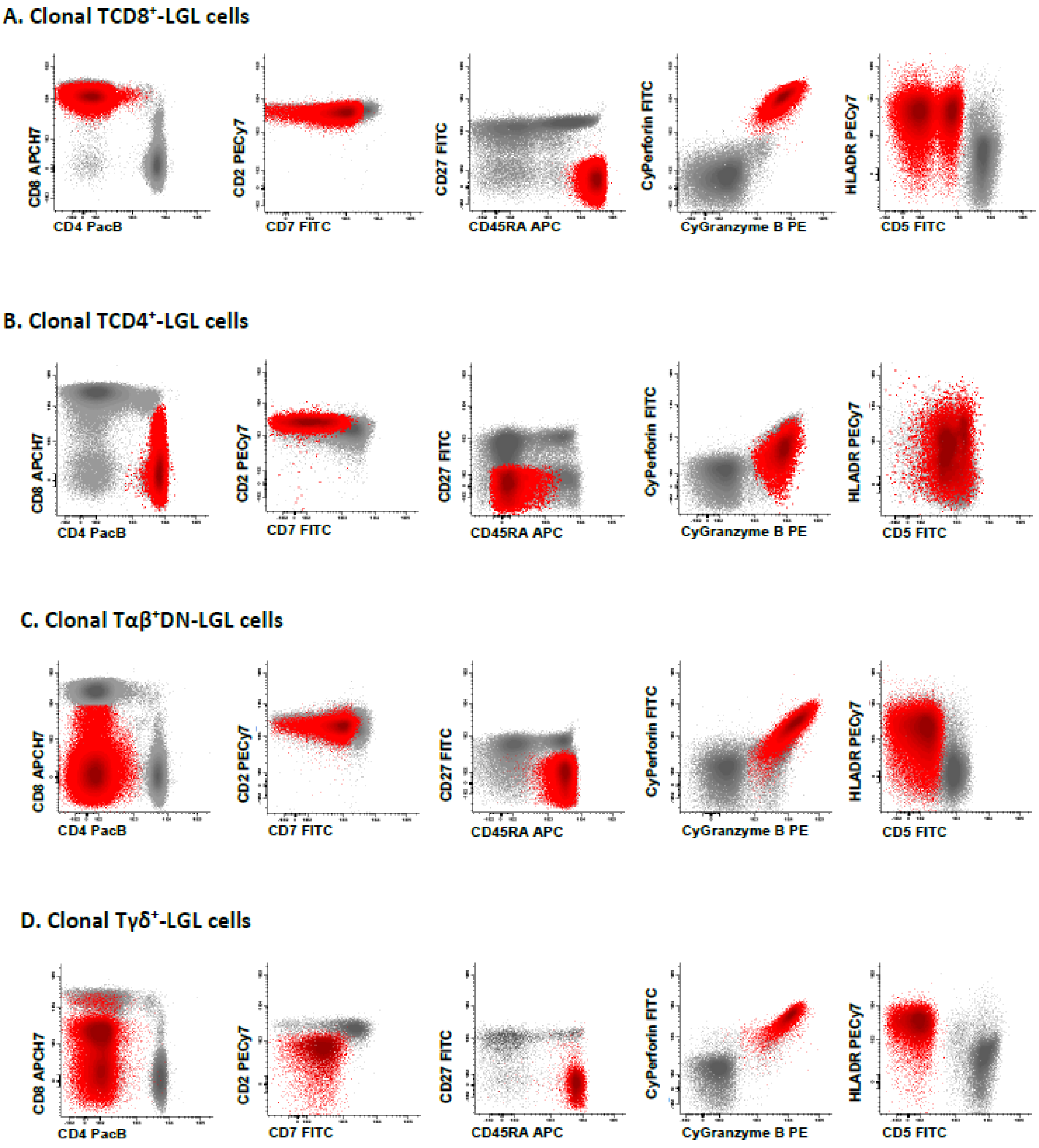

Figure S5. Phenotypic identification of distinct subtypes of aberrant clonal LGL of different T–cell lineages in T–LGLL patients. Red events correspond to the distinct populations of clonal T cells identified as different from all other normal residual T lymphocytes (gray dots). Selection of clonal T–cells was based on their CD2/CD7 (aberrant) profile and their cytotoxic (cyGranzyme^{+} and cyPerforin^{+}) effector memory/terminal effector (CD27^{-} and CD45RA$^{+lo/+}$) phenotype. In all panels, only T cells (CD3^{+} events) are displayed. Abbreviations (alphabetical order): LGL, large granular lymphocytes; LGLL, leukemia of LGL.

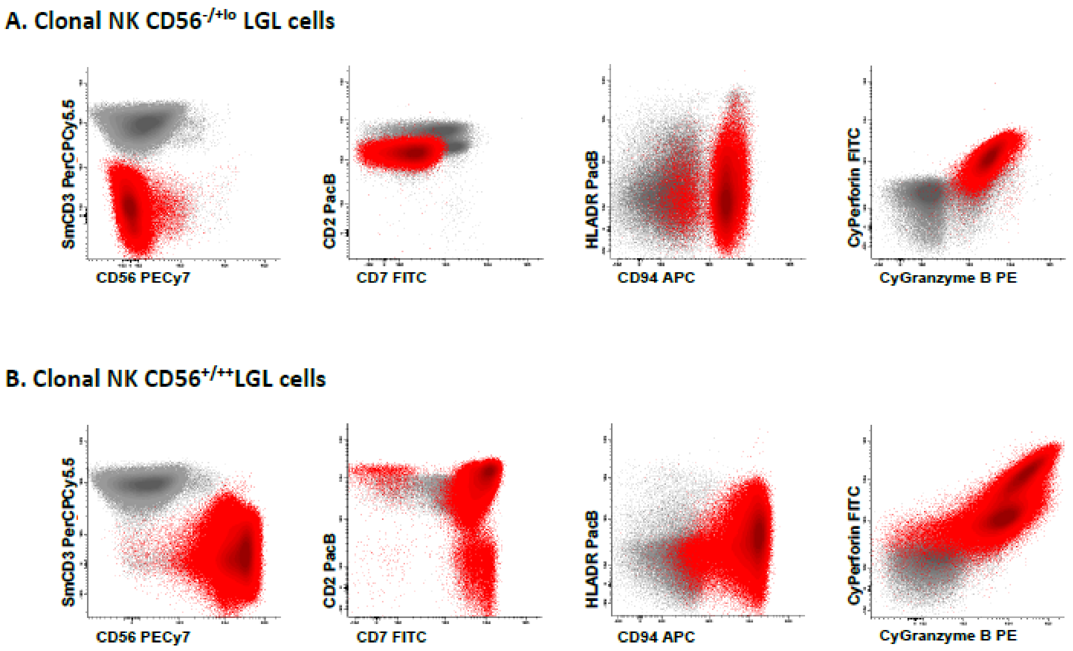

Figure S6. Phenotypic identification of distinct subtypes of aberrant clonal NK–cells in CLPD–NK patients. Red events correspond to distinct populations of (mono) clonal NK cells after their discrimination from all other T/NK lymphocytes (gray dots). Pathological NK–LGL cells were selected based on their CD2/CD7 aberrant phenotypic profile and their cytotoxic phenotype ($CD94^{+lo/+}$, $cyGranzyme^{+}$ and $cyPerforin^{+}$). In all panels, only T cells (CD3+ events) and NK cells are displayed. Abbreviations (alphabetical order): LGL, large granular lymphocytes; CLPD–NK: chronic lymphoproliferative disorders of NK cells.

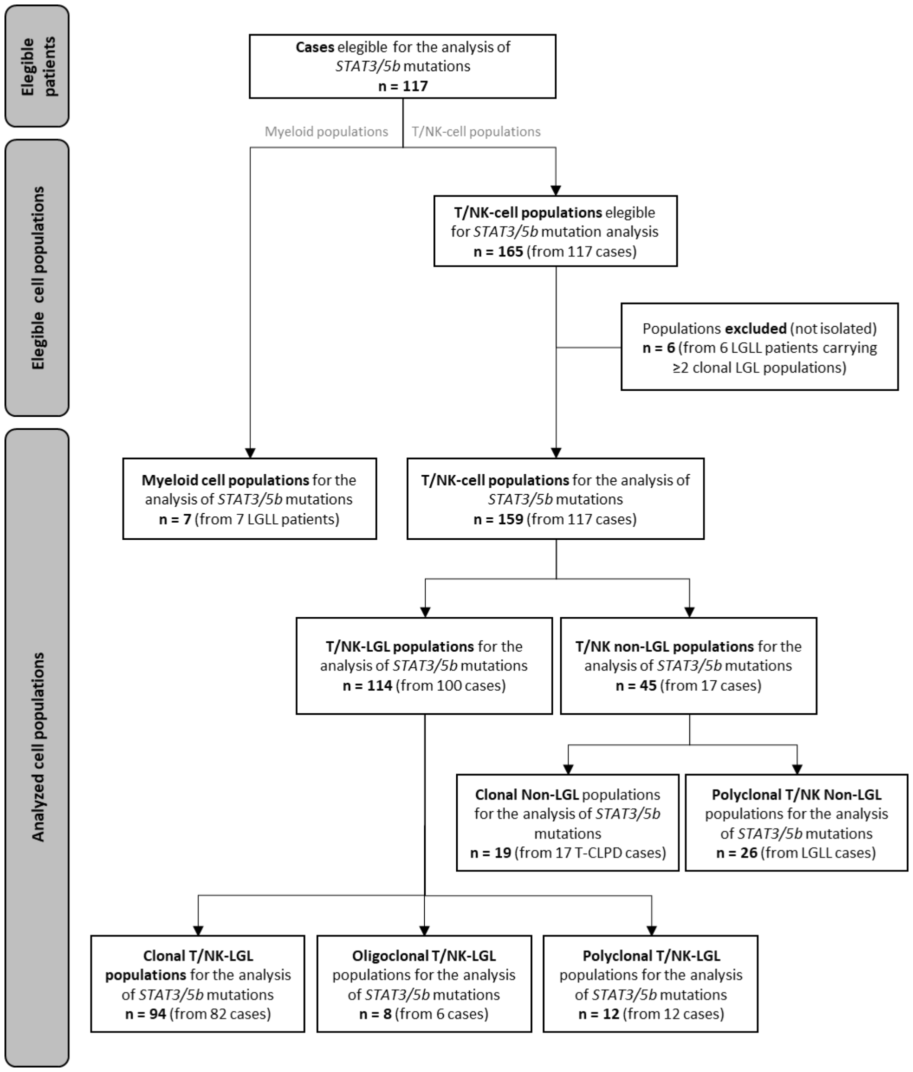

Figure S7. Flow–chart illustrating how the cases and cell populations were selected for the analysis of *STAT3* and *STAT5B* mutations.

Table S1. Distribution of *STAT3* or *STAT5B* –mutated T–LGL cell populations according to the T–cell receptor beta chain variable region (TCR–Vβ) or T–cell receptor gamma 9 and delta 2 chain region (TCR–Vγ9 and TCR–Vδ2) expressed.

TCR–V Family	N. *STAT3/5B*–Mutated/Total Clonal T–LGL Cell Populations	% of *STAT3/5B*–Mutated/Total Clonal T–LGL Cell Populations
TCR–Vβ1	1/1	100%
TCR–Vβ3	0/4	0%
TCR–Vβ5.1	2/8	25%
TCR–Vβ7.1	0/1	0%
TCR–Vβ7.2	0/2	0%
TCR–Vβ8	1/2	50%
TCR–Vβ11	0/1	0%
TCR–Vβ12	0/1	0%
TCR–Vβ13.1	3/8	38%
TCR–Vβ13.2	2/2	100%
TCR–Vβ13.6	1/5	20%
TCR–Vβ14	1/3	33%
TCR–Vβ16	0/1	0%
TCR–Vβ18	0/1	0%
TCR–Vβ22	1/3	33%
TCR–Vβ23	0/1	0%
TCR–Vβ NI	2/10	20%
TCR–$V\gamma9^{-}V\delta2^{-}$	1/8	13%
TCR–$V\gamma9^{+}V\delta2^{-}$	1/1	100%
TCR–$V\gamma9^{+}V\delta2^{+}$	0/2	0%

No cases with TCR–Vβ2, TCR–Vβ4, TCR–Vβ5.2, TCR–Vβ5.3, TCR–Vβ9, TCR–Vβ12, TCR–Vβ16, TCR–Vβ17, TCR–Vβ20, TCR–Vβ21.3 and TCR–$V\gamma9^{-}V\delta2^{+}$ were identified. Abbreviations (alphabetical order): N., number; NI, TCR–Vβ family not identified with the IOTest® TCR–Vβ Kit (Beckman Coulter).

Table S2. Distribution of normal PB leukocyte subsets in T–LGLL according to their *STAT3* or *STAT5B* mutational status.

**Cell Populations**	**Age–Matched HD (*n* = 628)**	**T–LGLL**		***p*–Value**
		**Wild–Type *STAT3* and *STAT5B* (*n* = 28)**	***STAT3* or *STAT5B* Mutated (*n* = 14)**	
Granulocytes (/μL)	**3977 (1678–8717)**	**2503 (298–10264)**	**1308 (46–6676)**	**≤0.01**[abc]
Neutrophils	**3709 (1563–8542)**	**2301 (263–10072)**	**1217 (39–4974)**	**≤0.02**[abc]
Eosinophils	**161 (0–876)**	**143 (8.4–1402)**	**30 (1.5–1250)**	**≤0.05**[abc]
Basophils	**41 (2.2–222)**	**51 (7.7–161)**	32 (4.3–452)	**0.05**[a]; 0.08[c]
Monocytes (/μL)	**320 (66–1247)**	**462 (23–1522)**	443 (150–972)	**0.0005**[a]
cMO	419 (215–1063)	406 (13–1426)	366 (128–838)	NS
ncMO	**67 (20–166)**	**38 (8.4–225)**	52 (4.5–149)	**0.01**[a]
Dendritic cells (/μL)	**24 (4.1–124)**	17 (0.48–54)	**8.2 (2–105)**	**0.007**[b]; 0.09[c]
Lymphocytes (/μL)	**1653 (0–5947)**	**2084 (943–7547)**	**1293 (553–4446)**	**≤0.03**[abc]
T lymphocytes (/μL)	**1188 (371–5298)**	**1672 (655–6469)**	**1097 (291–4039)**	**0.02**[ac]
TCD8$^+$	**386 (13–2199)**	463 (0–3133)	**278 (38–2120)**	**0.05**[b]; 0.08[c]
TCD4$^+$	**695 (106–3224)**	**970 (88–3994)**	**681 (227–2221)**	**≤0.03**[ac]
Tαβ$^+$DP	**8.6 (0–180)**	**28 (2.2–341)**	**24 (3.3–93)**	**≤0.001**[ab]
Tαβ$^+$DN	15 (2–117)	16 (2.2–71)	9.9 (5.3–77)	NS
Tγδ$^+$	**47 (1–765)**	**33 (0–598)**	**14 (0–265)**	**≤0.03**[bc]
NK cells (/μL)	**279 (0–1215)**	**150 (12–1383)**	**62 (7.4–408)**	**≤0.01**[abc]
CD56$^{-/+lo}$	5.2 (1.1–19)	2.6 (0.16–328)	2.6 (0–44)	NS
CD56$^+$	**350 (116–773)**	**144 (10–1372)**	**52 (0–403)**	**≤0.003**[abc]
CD56^{++}	4.5 (1.4–25)	6.8 (1.3–93)	6.6 (0.36–30)	NS
B lymphocytes (/μL)	141 (8.1–867)	169 (9.9–970)	161 (15–409)	NS
Plasma cells (/μL)	1.3 (0.104–14)	1.2 (0.091–28)	0.61 (0–14)	NS

Results expressed as median (range) values. In bold: statistically significant differences (*p*–value ≤ 0.05). [a]HD vs. WT cases; [b]HD vs. *STAT3*–mutated cases; [c]*STAT3*–mutated vs. WT cases. Abbreviations (alphabetical order): HD, healthy donors; c, classical; T–LGLL, T–cell large granular lymphocytic leukemia; MO, monocytes; nc, non–classical; NS, no statistically significant differences (*p*–value > 0.05); PB, peripheral blood; WT, wild–type.

Table S3. Distribution of normal PB leukocyte subsets in $TCD8^+$–LGLL according to their *STAT3* mutational status.

**Cell Populations**	**Age–Matched HD (*n* = 628)**	**$TCD8^+$–LGLL**		***p*–Value**
		**Wild–Type *STAT3* (*n* = 15)**	***STAT3* Mutated (*n* = 10)**	
Granulocytes (/µL)	**3977 (1678–8717)**	**2690 (298–10264)**	**1124 (46–2003)**	**≤0.02[abc]**
Neutrophils	**3709 (1563–8542)**	**2298 (263–10072)**	**1052 (39–1903)**	**≤0.01[abc]**
Eosinophils	**161 (0–876)**	**140 (20–1402)**	**30 (1.5–112)**	**≤0.003[bc]**
Basophils	41 (2.2–222)	40 (14–161)	28 (4.3–70)	0.07[b]; 0.06[c]
Monocytes (/µL)	**320 (66–1247)**	**401 (119–1223)**	472 (163–662)	**0.03[a]**; 0.08[b]
cMO	419 (215–1063)	342 (111–998)	388 (134–584)	NS
ncMO	**67 (20–166)**	**28 (8.4–225)**	52 (4.5–149)	**0.005[a]**
Dendritic cells (/µL)	**24 (4.1–124)**	**17 (1.2–54)**	**7.6 (2–32)**	**≤0.04[ab]**; 0.07[c]
Lymphocytes (/µL)	**1653 (0–5947)**	**2033 (1068–3362)**	**1257 (553–4446)**	**0.02[bc]**
T lymphocytes (/µL)	**1188 (371–5298)**	**1624 (780–2467)**	**1033 (291–4039)**	**≤0.02[ab]**
$TCD8^+$	**386 (13–2199)**	326 (0–773)	**278 (38–2120)**	**0.04[b]**
$TCD4^+$	**695 (106–3224)**	**962 (656–1853)**	**673 (227–1872)**	**≤0.01[ac]**
$T\alpha\beta^+DP$	**8.6 (0–180)**	**25 (2.2–341)**	**22 (3.3–93)**	**≤0.03[ab]**
$T\alpha\beta^+DN$	15 (2–117)	15 (2.2–71)	13 (5.3–77)	NS
$T\gamma\delta^+$	**47 (1–765)**	**29 (5.5–598)**	**15 (2.5–63)**	**≤0.04[bc]**
NK cells (/µL)	**279 (0–1215)**	**111 (33–1383)**	**62 (7.4–408)**	**≤0.002[ab]**; 0.06[c]
$CD56^{-/+lo}$	5.2 (1.1–19)	2.4 (0.56–328)	2.5 (0–44)	NS
$CD56^+$	**350 (116–773)**	**89 (25–1372)**	**54 (0–403)**	**≤0.03[abc]**
$CD56^{++}$	**4.5 (1.4–25)**	**8.5 (3.4–93)**	6.3 (0.46–30)	**0.05[a]**
B lymphocytes (/µL)	141 (8.1–867)	118 (9.9–303)	122 (15–409)	NS
Plasma cells (/µL)	1.3 (0.104–14)	0.86 (0.091–28)	0.58 (0–14)	NS

Results expressed as median (range) values. In bold: statistically significant differences (*p*–value ≤ 0.05). [a]HD vs. WT cases; [b]HD vs. *STAT3*–mutated cases; [c]*STAT3*–mutated vs. WT cases. Abbreviations (alphabetical order): HD, healthy donors; c, classical; T–LGLL, T–cell large granular lymphocytic leukemia; MO, monocytes; nc, non–classical; NS, no statistically significant differences (*p*–value > 0.05); PB, peripheral blood; WT, wild–type.

Table S4. Distribution of normal PB leukocyte subsets in Tγδ⁺–LGLL according to their *STAT3* mutational status.

Cell Populations	Age–Matched HD (*n* = 628)	Tγδ⁺–LGLL Wild–Type *STAT3* (*n* = 6)	Tγδ⁺–LGLL *STAT3* Mutated (*n* = 4)	*p*–Value
Granulocytes (/μL)	3977 (1678–8717)	3171 (643–4945)	2007 (343–6676)	0.08[b]
Neutrophils	**3709 (1563–8542)**	3045 (590–4702)	**1950 (295–4974)**	**0.05[b]**
Eosinophils	161 (0–876)	161 (8.4–291)	29 (20–1250)	NS
Basophils	41 (2.2–222)	55 (7.7–103)	32 (21–452)	NS
Monocytes (/μL)	320 (66–1247)	466 (23–1522)	453 (286–972)	0.1[a]
cMO	419 (215–1063)	403 (13–1426)	366 (241–838)	NS
ncMO	67 (20–166)	65 (9.8–96)	87 (45–133)	NS
Dendritic cells (/μL)	24 (4.1–124)	22 (0.48–39)	37 (5.6–105)	NS
Lymphocytes (/μL)	1653 (0–5947)	2733 (943–7546)	1117 (713–3696)	NS
T lymphocytes (/μL)	1188 (371–5298)	2224 (655–6469)	885 (291–3349)	0.08[a]
TCD8⁺	**386 (13–2199)**	**529 (493–2815)**	138 (38–1043)	**0.02[a]**; 0.1[b]
TCD4⁺	695 (106–3224)	1488 (88–3595)	546 (227–2221)	NS
Tαβ⁺DP	8.6 (0–180)	36 (5.5–79)	35 (3.3–67)	0.07[a]
Tαβ⁺DN	15 (2–117)	20 (5.4–42)	6.9 (6–60)	NS
Tγδ⁺	47 (1–765)	31 (0–121)	15 (0–265)	NS
NK cells (/μL)	279 (0–1215)	265 (12–1315)	94 (23–408)	0.06[b]
CD56⁻/⁺ˡᵒ	5.2 (1.1–19)	4.1 (0.16–6.5)	9 (0.94–18)	NS
CD56⁺	**350 (116–773)**	253 (10–1294)	**69 (21–403)**	**0.04[b]**
CD56⁺⁺	4.5 (1.4–25)	7.8 (1.3–15)	4.9 (0.36–8.8)	NS
B lymphocytes (/μL)	141 (8.1–867)	237 (13–748)	196 (15–209)	NS
Plasma cells (/μL)	1.3 (0.104–14)	2.6 (0.3–7.7)	1.1 (0.053–5)	NS

Results expressed as median (range) values. In bold: statistically significant differences (*p*–value ≤ 0.05). [a]HD vs. WT cases; [b]HD vs. *STAT3*–mutated cases. Abbreviations (alphabetical order): HD, healthy donors; c, classical; LGLL, leukemia of large granular lymphocytes; MO, monocytes; nc, non–classical; NS, no statistically significant differences (*p*–value > 0.05); PB, peripheral blood; WT, wild–type.

Table S5. Distribution of normal PB leukocyte subsets in CLPD–NK according to their STAT3 mutational status.

Cell Populations	Age–Matched HD (*n* = 628)	CLPD–NK Wild–Type *STAT3* (*n* = 4)	CLPD–NK *STAT3* Mutated (*n* = 5)	*p*–Value
Granulocytes (/μL)	**3977 (1678–8717)**	2672 (1180–6341)	**1078 (679–4625)**	**0.006[b]**
Neutrophils	**3709 (1563–8542)**	2512 (968–6112)	**1008 (671–4437)**	**0.006[b]**
Eosinophils	**161 (0–876)**	**139 (70–177)**	**6.1 (1.5–97)**	**≤0.03[bc]**

Basophils	41 (2.2–222)	59 (35–82)	32 (6.4–90)	NS
Monocytes (/μL)	320 (66–1247)	411 (359–645)	338 (198–1783)	NS (0.09[a])
cMO	419 (215–1063)	376 (301–534)	301 (198–1778)	NS
ncMO	**67 (20–166)**	63 (0–111)	**5.2 (0.49–37)**	**0.001[b]**
Dendritic cells (/μL)	**24 (4.1–124)**	17 (0–21)	**3.1 (0.7–13)**	**0.0008[b]**; NS (0.06[a])
Lymphocytes (/μL)	1653 (0–5947)	2283 (1253–3575)	1445 (553–3165)	NS
T lymphocytes (/μL)	1188 (371–5298)	1704 (983–2950)	1340 (502–3079)	NS
$TCD8^+$	386 (13–2199)	411 (150–1424)	404 (82–899)	NS
$TCD4^+$	**695 (106–3224)**	**1021 (793–1667)**	682 (285–1884)	**0.05[a]**
$T\alpha\beta^+DP$	**8.6 (0–180)**	34 (4.2–120)	**21 (7.2–200)**	**0.03[b]**
$T\alpha\beta^+DN$	15 (2–117)	17 (11–94)	28 (10–104)	NS
$T\gamma\delta^+$	47 (1–765)	14 (4.8–82)	30 (4.8–68)	NS
NK cells (/μL)	**279 (0–1215)**	139 (2–691)	**15 (0–46)**	**0.0001[b]**
$CD56^{-/+lo}$	5.2 (1.1–19)	6.7 (0–16)	0 (0–13)	NS
$CD56^+$	**350 (116–773)**	102 (0–664)	**0 (0–19)**	**0.0004[b]**
$CD56^{++}$	4.5 (1.4–25)	22 (2–31)	3 (0–46)	NS
B lymphocytes (/μL)	141 (8.1–867)	242 (60–540)	90 (44–251)	NS
Plasma cells (/μL)	1.3 (0.104–14)	1.4 (0.16–2.1)	1.4 (0.085–13)	NS

Results expressed as median (range) values. In bold: statistically significant differences (*p*–value ≤ 0.05). [a]HD vs. WT cases; [b]HD vs. *STAT3*–mutated cases; [c]*STAT3*–mutated vs. WT cases. Abbreviations (alphabetical order): HD, healthy donors; c, classical; CLPD–NK, chronic lymphoproliferative disorders of NK cells; MO, monocytes; nc, non–classical; NS, no statistically significant differences (*p*–value > 0.05); PB, peripheral blood; WT, wild–type.

Table S6. Clinical and biological features of clonal T–LGLL cases with wild–type (WT) vs. mutated *STAT3/5B*.

**Clinical and Biological Features**	**T–LGLL**		
	**Wild–Type *STAT3/5B* (*n* = 44)**	***STAT3/5B* Mutated (*n* = 23)**	***p*–Value**
Sex (male/female)*	22/22 (50%/50%)	10/13 (43%/57%)	NS
Age (years)	61 ± 17 (15–92)	62 ± 14 (40–90)	NS
Physical examination			
Organomegalies*[1]	5/32 (16%)	7/22 (32%)	NS
Skin lesions*	3/31 (10%)[2]	0/20 (0%)	NS
Peripheral blood cell counts			
Hemoglobin (g/dL)	13 ± 2.2 (8.3–18)	13 ± 2.1 (8.5–17)	NS
Platelets ($\times 10^9$/L)	**246 ± 74 (98–383)**	**201 ± 88 (25–421)**	**0.04**
Leukocytes ($\times 10^9$/L)	8.7 ± 4.5 (2.7–28)	6.8 ± 3.9 (0.9–17)	NS (0.08)
Clonal LGL cells ($\times 10^9$/L)	2.5 ± 2.9 (0.07–14)	2.8 ± 2.1 (0.5–5.9)	NS
Low–count clonal LGL lymphocytosis ($<0.5 \times 10^9$/L)*	14/39 (36%)	7/23 (30%)	NS
Very low–count clonal LGL lymphocytosis ($<0.1 \times 10^9$/L)*	5/39 (13%)	2/23 (9%)	NS
Cytopenias			
Anemia (≤10g/dL)*	5/39 (13%)	3/22 (14%)	NS
Thrombocytopenia ($\leq 100 \times 10^9$/L)*	1/38 (3%)	2/22 (9%)	NS
Neutropenia ($\leq 1 \times 10^9$/L)*	**5/39 (13%)**	**9/23 (39%)**	**0.02**
Severe Neutropenia ($\leq 0.5 \times 10^9$/L)*	**0/39 (0%)**	**4/23 (17%)**	**0.02**
Other associated diseases			
Other clonal/neoplastic diseases*	9/30 (30%)	3/20 (15%)	NS
Autoimmune diseases* (including cytopenias)	14/32 (44%)	15/23 (65%)	NS (0.1)
Autoimmune diseases* (other than cytopenias)	4/31 (13%)	7/23 (30%)	NS
Other diseases*	8/31 (26%)	9/20 (45%)	NS
Outcome and follow–up			
Need for LGLL therapy*[3]	**3/38 (8%)**	**9/20 (45%)**	**0.002**
Time to LGLL therapy (months)[#]	**Not reached**[$]	**72 (1–179)**	**0.001**
Disease Progression*	2/32 (6%)	1/19 (5%)	NS
Deaths* (overall deaths)	6/38 (16%)	2/20 (10%)	NS
Deaths*[4]	0/38 (0%)	1/19 (5%)	NS

Results expressed as mean ± standard deviation (SD) (and range), * as number of cases (percentage) or [#] as median (95% confidence interval). In bold: statistically significant differences (*p*–value ≤ 0.05). [$]After a median follow at of 183. [1]Adenopathy, splenomegaly and/or hepatomegaly. [2]Scleroderma. [3]In all cases, treatment was administered because of the presence of cytopenias and/or other associated autoimmune diseases. [4]All deaths were due to complications derived from the associated autoimmune disease. Abbreviations (alphabetical order): LGLL, leukemia of large granular lymphocytes; NS, no statistically significant differences (*p*–value > 0.05). T–LGLL, T–cell large granular lymphocytic leukemia.

Table S7. Clinical and biological features of TCD8[+]–LGLL according to their *STAT3* mutational status and the presence of bi(multi) vs. monoclonal LGL populations.

Clinical and biological features	Bi(multi) vs. monoclonal TCD8+–LGLL				
	Wild–Type *STAT3*		*STAT3* mutated		
	Bi(multi)clonal (*n* = 4)	Monoclonal (*n* = 12)	Bi(multi)clonal (*n* = 5)	Monoclonal (*n* = 9)	*p*–Value
Sex (male/female)*	3/1 (75%/25%)	7/5 (58%/42%)	2/3 (40%/60%)	5/4 (56%/44%)	NS
Age (years)	75 ± 14 (54–85)	61 ± 21 (15–92)	66 ± 19 (44–90)	62 ± 14 (49–83)	NS
Physical examination					
Organomegalies*[1]	0/4 (0%)	1/9 (11%)	2/5 (40%)	3/9 (33%)	NS
Peripheral blood cell counts					
Hemoglobin (g/dL)	14 ± 1.1 (14–16)	13 ± 3 (8.3–18)	13 ± 1 (11–14)	14 ± 2.3 (9.7–17)	NS
Platelets (×10^9/L)	239 ± 110 (98–363)	244 ± 76 (170–383)	216 ± 136 (61–421)	185 ± 63 (121–302)	NS
Leukocytes (×10^9/L)	9.3 ± 3.3 (6.1–13)	8.8 ± 6.8 (3.1–28)	6.3 ± 3.5 (2.7–12)	7.2 ± 3.2 (3.1–13)	NS
Clonal LGL cells (×10^9/L)	2.2 ± 2.1 (0.5–5.2)	3.5 ± 4.5 (0.4–14)	2.2 ± 1.6 (1.2–4.5)	3.1 ± 2.3 (0.6–5.4)	NS
Leukocytosis (>10 × 10^9/L)*	2/4 (50%)	2/10 (20%)	1/5 (20%)	1/8 (13%)	NS
Lymphocytosis (>3.5 × 10^9/L)*	3/4 (75%)	5/10 (50%)	2/5 (40%)	4/8 (50%)	NS
Lymphocytosis (>5 × 10^9/L)*	3/4 (75%)	3/10 (30%)	2/5 (40%)	4/8 (50%)	NS
Cytopenias					
Anemia (≤10g/dL)*	0/4 (0%)	1/10 (10%)	0/5 (0%)	0/8 (0%)	NS
Thrombocytopenia (≤100 × 10^9/L)*	1/4 (25%)	0/9 (0%)	1/5 (20%)	0/8 (0%)	NS (0.1)[a]
Neutropenia (≤1 × 10^9/L)*	0/4 (0%)	3/10 (%)	2/5 (40%)	3/8 (38%)	NS
Severe Neutropenia (≤0.5 × 10^9/L)*	0/4 (0%)	0/10 (0%)	0/5 (0%)	1/8 (13%)	NS
Other associated diseases					
Other clonal/neoplastic diseases*	0/3 (0%)	2/10 (20%)	1/5 (20%)	2/8 (25%)	NS
Autoimmune diseases* (including cytopenias)	2/3 (67%)	6/11 (55%)	3/5 (60%)	5/9 (55%)	NS
Autoimmune diseases* (other than cytopenias)	1/3 (33%)	1/11 (9%)	2/5 (40%)	2/9 (22%)	NS
Other diseases*	0/3 (0%)	2/10 (20%)	2/5 (40%)	2/8 (25%)	NS
Outcome and follow–up					
Need for LGLL therapy*[2]	1/4 (25%)	1/11 (9%)	3/4 (75%)	3/9 (33%)	NS
Time to LGLL therapy (months)#	55 (24–86)	65 (53–77)	17 (1–42)	70 (39–101)	NS (0.08)
Disease progression*	0/4 (0%)	1/9 (11%)	1/4 (25%)	0/9 (0%)	NS
Deaths* (overall deaths)	1/4 (25%)	2/11 (20%)	1/4 (25%)	1/9 (11%)	NS
Deaths*[3]	0/4 (0%)	0/11 (20%)	1/4 (25%)	0/8 (0%)	NS

Results expressed as mean ± standard deviation (SD) (and range), * as number of cases (percentage) or # as mean (95% confidence interval) since median values were not reached. [1]Adenopathy, splenomegaly and/or hepatomegaly. [2]In all cases, treatment was administered because of the presence of cytopenias and/or other associated autoimmune diseases. [3]Death was due to complications derived from the associated autoimmune disease. [a]Biclonal cases vs. monoclonal cases. Abbreviations (alphabetical order): LGLL, leukemia of large granular lymphocytes; NS, no statistically significant differences (*p*–value > 0.05); T–LGLL, T–cell large granular lymphocytic leukemia.

Table S8. Clinical and biological features of clonal CLPD–NK cases with wild–type (WT) vs. mutated *STAT3*.

Clinical and biological features	CLPD–NK

	**Wild–Type *STAT3* (*n* = 10)**	***STAT3* mutated (*n* = 6)**	***p*–Value**
Sex (male/female)*	**1/9 (10%/90%)**	**4/2 (67%/33%)**	0.04[1]
Age (years)	51 ± 26 (4–83)	76 ± 12 (56–89)	NS (0.06)
Physical examination			
Organomegalies*[2]	0/2 (0%)	3/6 (50%)	NS
Skin lesions*	1/2 (50%)[3]	0/5 (0%)	NS
Peripheral blood cell counts			
Hemoglobin (g/dL)	**13 ± 1.1 (11–14)**	**11 ± 2.6 (6.3–13)**	**0.04**
Platelets (x10^9/L)	230 ± 62 (150–321)	185 ± 104 (61–346)	NS
Leukocytes (x10^9/L)	16 ± 16 (7.3–55)	12 ± 13 (2.7–36)	NS
Clonal LGL cells (x10^9/L)	16 ± 23 (2.4–50)	7.3 ± 13 (0.6–30)	NS
Leukocytosis (>10x10^9/L)*	4/8 (50%)	3/6 (50%)	NS
Lymphocytosis (>3.5x10^9/L)*	**8/8 (100%)**	**3/6 (50%)**	**0.05**
Lymphocytosis (>5x10^9/L)*	5/8 (63%)	2/6 (33%)	NS
Cytopenias			
Anemia (≤10g/dL)*	0/7 (0%)	2/6 (33%)	NS
Thrombocytopenia (≤100x10^9/L)*	0/7 (0%)	2/6 (33%)	NS
Neutropenia (≤1x10^9/L)*	1/8 (13%)	1/6 (17%)	NS
Other associated diseases			
Other clonal/neoplastic diseases*	1/2 (50%)	1/6 (17%)	NS
Autoimmune diseases* (including cytopenias)	2/3 (67%)	4/6 (67%)	NS
Autoimmune diseases* (other than cytopenias)	1/2 (50%)	0/6 (0%)	NS
Other diseases*	2/2 (100%)	1/4 (25%)	NS
Outcome and follow–up			
Need for LGLL therapy*[4]	1/6 (17%)	4/5 (80%)	NS (0.08)
Time to LGLL therapy (months)#	**88 (56–119)**	**6 (1–15)**	**0.05**
Disease progression*	**0/4 (0%)**	**3/4 (75%)**	NS (0.1)
Deaths* (overall deaths)	2/6 (33%)	3/5 (60%)	NS
Deaths*[5]	1/6 (17%)	2/5 (40%)	NS

Results expressed as mean ± standard deviation (SD) (and range), * as number of cases (percentage) or # as mean (95% confidence interval) since median values were not reached. In bold: statistically significant differences (*p*–value ≤ 0.05). [1]Selection bias, due to the absence of an assay to confirm clonality in male patients but the presence of *STAT3* mutation; the only male case included in the WT group had an aberrant NK–cell phenotype ($CD2^{-}CD94^{++}HLADR^{++}$), previously found to be systematically associated with monoclonality [14]. [2]Adenopathy, splenomegaly and/or hepatomegaly. [3]Scleroderma. [4]In all cases, treatment was administered because of the presence of cytopenias and/or other associated autoimmune diseases. [5]All deaths were due to complications derived from the

associated autoimmune disease. Abbreviations (alphabetical order): LGLL, leukemia of large granular lymphocytes; CLPD–NK, chronic lymphoproliferative disorder of NK cells; NS, no statistically significant differences (*p*–value > 0.05); WT, wild–type.

Table S9. Distribution of LGLL cases and non–LGL CLPD patients (*n* = 117) included in this study and the corresponding populations of LGL identified (*n* = 165) classified according to their phenotypic profile and (mono vs. oligo/poly) clonal nature.

**Distribution of Cases and Populations Included**		**LGL Lymphocytosis**				**Non–LGL Cell Populations From CLPD**		
		**Clonal**[¥]	**Oligoclonal**	**Polyclonal**	**TOTAL LGL**	**Clonal (CLPD)***	**Polyclonal****	**TOTAL Non–LGL**
		***n* = 82 (100)**	***n* = 6 (8)**	***n* = 12 (12)**	***n* = 100 (120)**	***n* = 17 (19)**	***n* = NA (26)**	***n* = 17 (45)**
**T–cell lineage**<br>*n* = 94<br>–77 LGL and 17 non–LGL–<br>(*n* = 141 populations)	TCD8$^+$	33 (50)<br>*40% (50%)*	4 (5)<br>*67% (63%)*	4 (4)<br>*34% (34%)*	**41 (59)**<br>***41% (49%)***	2 (3)	(11)	**2 (14)**
	LGL TCD4$^+$	14 (15)<br>*17% (15%)*			**14 (15)**<br>***14% (12%)***			
	Non–LGL TCR$\alpha\beta^+$CD4$^+$CD8$^-$					11 (11)	(12)	**11 (23)**
	T$\alpha\beta^+$DP	1 (1)<br>*1% (1%)*			**1 (1)**<br>***1% (1%)***		(1)	**(1)**
	T$\alpha\beta^+$DN	2 (2)<br>*2% (2%)*			**2 (2)**<br>***2% (2%)***	1 (1)	(1)	**1 (2)**
	T$\gamma\delta^+$	16 (16)<br>*20% (16%)*	2 (3)<br>*33% (37%)*	1 (1)<br>*8% (8%)*	**19 (20)**<br>***19% (16%)***	3 (4)		**3 (4)**
Subtotal		*66 (84)*	*6 (8)*	*5 (5)*	***77 (97)***	*17 (19)*	*0 (25)*	***17 (44)***
**NK–cell lineage**<br>*n* = 23<br>(*n* = 24 populations)	CD56$^{-/+lo}$	7 (7)<br>*9% (7%)*		1 (1)<br>*8% (8%)*	**8 (8)**<br>***8% (7%)***			
	CD56$^+$	7 (7)<br>*9% (7%)*		6 (6)<br>*50% (50%)*	**13 (13)**<br>***13% (11%)***			
	CD56^{++}	2 (2)<br>*2% (2%)*			**2 (2)**<br>***2% (2%)***		(1)	**(1)**
Subtotal		*16 (16)*	*0 (0)*	*7 (7)*	***23 (23)***	*0 (0)*	*0 (26)*	***0 (1)***

Results expressed as number of cases (number of cell populations) studied and their percentage (in italic) per LGL Lymphocytosis category. Empty cells mean no cases and no cell populations. In bold: total LGL and non-LGL cases and populations per category [¥]A more in–depth study of all LGL populations was carried out in 59/82 monoclonal cases, from which 14/59 clonal LGL cases (24%) showed ≥2 different populations of expanded/aberrant LGL cells, and they were classified within the phenotypic group corresponding to the major cell population, as follows: i) 6/14 cases were classified as TCD8$^+$ (2 cases had two different clonal populations of TCD8$^+$ cells; 2 cases had three different clonal populations of TCD8$^+$ cells; 1 case had four different clonal populations of TCD8$^+$ cells; and 1 case had one clonal population of TCD8$^+$ cells coexisting with a TCD4$^+$ population; ii) 4/14 cases classified as T$\gamma\delta^+$ category (all showing one clonal T$\gamma\delta^+$ cell population coexisting with another TCD8$^+$ cell population); iii) 2/14 cases were classified as TCD4$^+$ (both of them displaying an additional clonal TCD8$^+$ cell population); iv) 1/14 cases classified as T$\alpha\beta^+$DN (one clonal T$\alpha\beta^+$DN population coexisted with another TCD8$^+$ cell clonal population); and v) 1/14 cases belonged to the CD56$^{-/+lo}$ NK–

cell category (with an additional $TCD8^+$ cell clonal population). A total of 8 clonal LGL populations out of the 14 multiclonal cases were sorted for further analysis, while the remaining 6 T–LGL populations found to be clonal by phenotype could not be purified. *Monoclonal non–LGL cases (n = 17) correspond to: 7 peripheral T–cell lymphoma not otherwise specified; 3 T–cell prolymphocytic leukemia; 2 hepatosplenic T–cell lymphomas; 1 Sézary syndrome; 1 adult T–cell leukemia/lymphoma; 1 $CD3^-CD4^+$ lymphoid variant of hypereosinophilic syndrome; 1 intestinal T–cell lymphoma; 1 hydroa vacciniforme–like lymphoproliferative disorder. In each case, a single clonal non–LGL population was identified and purified for further analyses, except in a patient diagnosed with Sézary syndrome and a hepatosplenic T–cell lymphoma case, in which two different clonal T–cell populations were identified and purified. **Phenotypically normal residual T/NK–cell populations (polyclonal) from patients with clonal T–cell expansions (control group). In addition to normal residual T/NK–cell populations, a total of 7 myeloid cell populations (i.e., neutrophils) from patients with different clonal T–LGL disorders were also screened for the presence of *STAT3* and *STAT5B* mutations. Abbreviations (alphabetical order): CLPD, chronic lymphoproliferative disorder; LGL, large granular lymphocytes; NA, not applicable.

Table S10. Panels of fluorochrome–conjugated antibodies (specificities and sources) used for sequential immunophenotypic analyses of LGLL by flow cytometry.

Suppl. Panel A. LST (EuroFlow Lymphocytosis Screening Tube)[1]

**PacB**	**OC515***	**FITC**	**PE**	**PerCPCy5.5**	**PECy7**	**APC**	**APCH7**
CD20<br>CD4	CD45	CD8<br>Anti–Igλ	CD56<br>Anti–Igκ	CD5	CD19<br>Anti–TCRγδ	SmCD3	CD38

**Marker**	**Fluorochrome**	**Clone**	**Manufacturer**
SmCD3	APC	SK7	BD
CD4	PacB	RPA–T4	BioLegend
CD5	PerCPCy5.5	L17F12	BD
CD8	FITC	UCH–T4	Cytognos
CD19	PECy7	J3–119	Beckman Coulter
CD20	PacB	2H7	BioLegend
CD38	APCH7	HB7	BD
CD45	OC515	GA90	Cytognos
CD56	PE	C5.9	Cytognos
Igκ	PE	Polyclonal	Cytognos
Igλ	FITC	Polyclonal	Cytognos
TCRγδ	PECy7	11F2	BD

Suppl. Panel B. Immunophenotypic panel assessed to T–cell clonality of TCR–Vβ (Panel B.1) and both TCR–Vγ and Vδ cells (Panel B.2)

Suppl. Panel B.1. TCR–Vβ clonality assessment using the *IOTest® Beta Mark TCR Vβ Repertoire Kit (Beckman Coulter)*[2]

**Tube**	**PacB**	**OC515***	**FITC**	**PE**	**PerCPCy5.5**	**PECy7**	**APC**	**APCH7**
A–H	CD4	–	TCR–Vβ "a"<br>TCR–Vβ "b"	TCR–Vβ "b"<br>TCR–Vβ "c"	SmCD3	–	–	CD8

**"Tube"**		**Marker**	**Fluorochrome**	**Clone**	**Manufacturer**
A	a	TCR–Vβ 5.3	PE	3D11	Beckman Coulter

	b	TCR–Vβ 7.1	PE + FITC	ZOE	Beckman Coulter
	c	TCR–Vβ 3	FITC	CH92	Beckman Coulter
B	a	TCR–Vβ 9	PE	FIN9	Beckman Coulter
	b	TCR–Vβ 17	PE + FITC	E17.5F3	Beckman Coulter
	c	TCR–Vβ 16	FITC	TAMAYA1.2	Beckman Coulter
C	a	TCR–Vβ 18	PE	BA62.6	Beckman Coulter
	b	TCR–Vβ 5.1	PE + FITC	IMMU157	Beckman Coulter
	c	TCR–Vβ 20	FITC	ELL1.4	Beckman Coulter
D	a	TCR–Vβ 13.1	PE	IMMU222	Beckman Coulter
	b	TCR–Vβ 13.6	PE + FITC	JU74.3	Beckman Coulter
	c	TCR–Vβ 8	FITC	56C5.2	Beckman Coulter
E	a	TCR–Vβ 5.2	PE	36213	Beckman Coulter
	b	TCR–Vβ 2	PE + FITC	MPB2D5	Beckman Coulter
	c	TCR–Vβ 12	FITC	VER2.32	Beckman Coulter
F	a	TCR–Vβ 23	PE	AF23	Beckman Coulter
	b	TCR–Vβ 1	PE + FITC	BL37.2	Beckman Coulter
	c	TCR–Vβ 21.3	FITC	IG125	Beckman Coulter
G	a	TCR–Vβ 11	PE	C21	Beckman Coulter
	b	TCR–Vβ 22	PE + FITC	IMMU546	Beckman Coulter
	c	TCR–Vβ 14	FITC	CAS1.1.3	Beckman Coulter
H	a	TCR–Vβ 13.2	PE	H132	Beckman Coulter
	b	TCR–Vβ 4	PE + FITC	WJF24	Beckman Coulter
	c	TCR–Vβ 7.2	FITC	ZIZOU4	Beckman Coulter

Suppl. Panel B.2. Assessment of TCR–Vγ and Vδ clonality[2]

Tube	PacB	OC515*	FITC	PE	PerCPCy5.5	PECy7	APC	APCH7
1	CD4	–	TCR–γ9	–	SmCD3	–	–	CD8
2	CD4	–	TCR–δ2	–	SmCD3	–	–	CD8

Marker	Fluorochrome	Clone	Manufacturer
TCR–Vγ9	FITC	IMMU360	Beckman Coulter
TCR–Vδ2	FITC	IMMU389	Beckman Coulter

Suppl. Panel C. EuroFlow panels for characterization/classification of T–cell (Panel C.1) and NK–cell (Panel C.2) chronic lymphoproliferative disorders (CLPD)

Suppl. Panel C.1. EuroFlow T–cell CLPD panel

Tube	PacB	OC515*	FITC	PE	PerCPCy5.5	PECy7	APC	APCH7
1	CD4	CD45	CD7	CD26	SmCD3	CD2	CD28	CD8
2	CD4	CD45	CD27	CD197	SmCD3	CD45RO	CD45RA	CD8
3	CD4	CD45	CD5	CD25	SmCD3	HLADR	CyTCL1	CD8
4	CD4	CD45	CD57	CD30	SmCD3	–	CD11c	CD8
5	CD4	CD45	CyPER	CyGRA	SmCD3	CD16	CD94	CD8

6	CD4	CD45	–	CD279	SmCD3	–	–	CD8

**Marker**	**Fluorochrome**	**Clone**	**Manufacturer**
CD2	PECy7	L303.1	BD
SmCD3	PerCPCy5.5	SK7	BD
CD4	PacB	RPA–T4	BioLegend
CD5	FITC	L17F12	BD
CD7	FITC	4H9	BD
CD8	APCH7	SK1	BD
CD11c	APC	S–HCL–3	BD
CD16	PECy7	3G8	BD
CD25	PE	2A3	BD
CD26	PE	L272	BD
CD27	FITC	L128	BD
CD28	APC	CD28.2	BD
CD30	PE	BerH8	BD
CD45	OC515	GA90	Cytognos
CD45RA	APC	HI100	BD
CD45RO	PECy7	UCHL1	BD
CD57	FITC	HNK–1	BD
CD94	APC	HP–3D9	BD
CD197	PE	150503	R&D
CD279	PE	MIH4	BD
CyGranzyme B	PE	CLB–GB11	Sanquin
CyPerforin	FITC	δG9	BD
CyTCL1	APC	eBio1–21	eBioscience
HLADR	PECy7	L243	BD

Suppl. Panel C.2. EuroFlow NK–cell CLPD panel

Tube	PacB	OC515*	FITC	PE	PerCPCy5.5	PECy7	APC	APCH7
1	CD2	CD45	CD7	CD26	SmCD3	CD56	CD5	CD19
2	CD16	CD45	CD57	CD25	SmCD3	CD56	CD11c	CD19
3	HLAD R	CD45	CyPER	CyGRA	SmCD3	CD56	CD94	CD19

Marker	Fluorochrome	Clone	Manufacturer
CD2	PacB	TS1/8	Biolegend
SmCD3	PerCPCy5.5	SK7	BD
CD5	APC	L17F12	BD
CD7	FITC	4H9	BD
CD11c	APC	S–HCL–3	BD
CD16	PacB	3G8	BD

CD19	APCH7	SJ25C1	BD
CD25	PE	2A3	BD
CD26	PE	L272	BD
CD45	OC515	GA90	Cytognos
CD56	PECy7	N901	Beckman Coulter
CD57	FITC	HNK–1	BD
CD94	APC	HP–3D9	BD
CyGranzyme B	PE	CLB–GB11	Sanquin
CyPerforin	FITC	δG9	BD
HLADR	PacB	L243	Biolegend

For all panels, "stain & lyse" EuroFlow SOPs were used [56]. Combined staining for surface antigens and intracellular molecules was performed using the Fix & Perm™ reagent kit (Thermo Fisher Scientific, Waltham, MA), according to the recommendations of the manufacturer and the EuroFlow SOPs. *May also be Pacific Orange (PacO). [1] Since 2016 *OneFlow™ LST (Becton/Dickinson Biosciences) was* used for the staining of the samples. [2] In specific cases, additional markers were also included in the empty fluorescence channels ("–"), to better identify aberrant cells (i.e., CD5).Abbreviations (alphabetical order): APC, allophycocyanin; APCH7, allophycocyanin Hilite®7; BD, Becton/Dickinson Biosciences; Cy, cytoplasmic; FITC, fluorescein isothiocyanate; Ig, immunoglobulin; OC515, Orange Cytognos™ 515; PacB, Pacific Blue™; PE, phycoerythrin; PECy7, phycoerythrin–cyanin 7; PerCPCy5.5, peridinin–chlorophyll–cyanin 5.5; Sm, surface membrane; TCR, T–cell receptor.

Table 11. *STAT3* and *STAT5B* primers and temperature conditions used for PCR–based gene mutation analysis.

Gene	Location	Forward Primer	Reverse Primer	Amplicon	Annealing Temperature
STAT3	Exon 19–20	5′–CCAGTAGGACCTGCCTGAAG–3′	5′–GCAAATGTGTTTTGCGAGTC–3′	611 bp	55 °C
	Exon 21	5′–TCTTTCCTTCCCATGTCCTG–3′	5′–CAAGGATCCCAAAATTTCCA–3′	304 bp	50 °C
STAT5B	Exon 16	5′–TGTTGGGGTTTTAAGATTTCC–3′	5′–CAAATCAGAATGCGAACATTG–3′	266 bp	54 °C

Abbreviations (alphabetical order): bp, base pair; PCR, polymerase chain reaction.
